# Preparation, physical, optical, ESR and γ-ray attenuation efficacy investigation of copper oxide/silver borosilicate glass

**DOI:** 10.1038/s41598-024-75017-9

**Published:** 2024-10-25

**Authors:** Ahmed M. A. El-Seidy, O. I. Sallam, Islam M. Nabil, Yasser S. Rammah, Mohamed S. El-Okaily, Heba Alshater

**Affiliations:** 1 Inorganic Chemistry Department, Advanced Materials Technology & Mineral Resources Research Institute, National Research Centre, El-bohouth St., Dokki, P.O. 12622 Cairo, Egypt; 2 Glass Lab, Radiation Chemistry Department, National Center for Radiation Research and Technology, Egyptian Atomic Energy Authority (EAEA), Cairo, Egypt; 3 Physics Department, Faculty of Science, Fayoum University, Fayoum, Egypt; 4 Department of Physics, Faculty of Science, Menoufia University, Shebin El-Koom, 32511 Menoufia, Egypt; 5 Pharos University in Alexandria, Canal El Mahmoudia Street, Beside Green Plaza Complex, 21648 Alexandria, Egypt; 6 Refractories, Ceramics & Building Materials Department (Biomaterials Group), Advanced Materials Technology & Mineral Resources Research Institute, National Research Centre, Cairo, Egypt; 7 Forensic Medicine and Clinical Toxicology Department, Menoufia University Hospital, Shebin El-Koom, 32511 Menoufia, Egypt

**Keywords:** Ag/CuO nanocomposites, CuO-Nps, XRD, SEM, Borosilicate glass, Shielding parameter, Materials science, Optics and photonics, Chemistry, Inorganic chemistry

## Abstract

The sonication method was used to prepare a new set of CuO and CuO/Ag nanocomposites. The particle size was estimated using XRD and HR-TEM while the morphology of the nanoparticles was investigated with SEM. The average particle sizes of CuO and Ag falls in the 29.32–35.80 nm and 35.13–45.95 nm ranges, respectively. XRD declared that CuO has the space groups C 1 c 1 (9) and C 1 2/c 1 (15), while silver has space group F m -3 m (225). XPS analysis indicated the presence of Ag as Ag^0^ and Cu as Cu^2+^. Nano-oxide and nanocomposites were used to synthesis CuO and CuO/Ag doped lithium–zinc borosilicate glass. Physical parameters of the glass samples were calculated including density, $\hbox {V}_{m}$, $\hbox {V}_{o}$, $\hbox {V}_{m}^{B}$, OPD, $\hbox {d}_{B-B}$, $\hbox {n}_{b}$, and N, $\hbox {R}_{p}$, and $\hbox {R}_{i}$ depending on Ag and CuO mole fractions. The physical properties of glass indicated an increase in density and an initial expansion in glass structural network with the addition of silver metal due to its larger size followed by a compression as its molar ratio increase due to its higher $\hbox {C}_{no}$ . XRD measurements were reported for the glass samples doped with nanoparticles, proving the amorphous phase. ESR measurements were determined for all glass samples to detect the nature of the doped nanoparticles when incorporated inside a glassy matrix where CuO was found as tetragonal in octahedral sites and silver can be transformed after melting inside the glass matrix into Ag^+^ to form more stable $\hbox {Ag}^{x+}_{y}$ clusters.The fabricated sample of CuAgB-4 with significant nano silver doping (7.32% mol) has the maximum LAc and effective atomic number. Nano silver content increases the $\gamma$-RdSg in the lithium–zinc borosilicate glasses.

## Introduction

Gamma radiation is a high-energy photons electromagnetic radiation. It provides substantial issues in a variety of disciplines, including nuclear power, medical imaging, basic science, biological research and therapy applications^1^. Despite the amazing advantages of $\gamma$-beams, it reacts negatively to living tissue. Being high ionizing radiation enable it to enters and travel through the body, causing ionization and tissue damage. Furthermore, they have the ability to interact with inanimate materials, which can cause the particles to dissolve their bonds and release free radicals^2^. Therefore, creating a suitable and effective shielding material is necessary to protect the environment and human beings from its dangerous effects. The weight, space, cost and attenuation or absorption capabilities of materials used for radioactive protection are the main factors that make it difficult for researchers to create and develop suitable shielding materials^3^. The conventional materials used for shielding, such as lead and concrete, possess some drawbacks, including their elevated density, toxicity, challenging shape and susceptibility to cracking^4^. Despite the fact that silver has many uses, its high cost limits its utilization. Copper is therefore a cheaper metal that can be added to silver. Owing to their special characteristics, copper–silver nanoparticles have become a promising option for shielding against gamma radiation. Since copper and silver have high atomic numbers and excellent electrical conductivity, they are good at absorbing gamma radiation. The performance of these metallic nanoparticles as a radiation shield is improved when they are included in a matrix^5^. High-boron lithium-borosilicate glass has attracted a lot of interest in material research because of its potential for technology. The different compositions of these glasses are being investigated for their potential use in a range of applications, such as photonics, electronic displays, network modifications, sensors^6^, tissue engineering^7^, neutron shielding materials^8^. Lithium-borate glasses exhibit distinct physical characteristics, including elevated ionic conductivity and notable thermal expansion coefficient^9^, low melting temperature^10^. Consequently, their utility in diverse domains is increasing with time. The fundamental constituents of borate compounds consist of BO_3_ and BO_4_ groups^10^. The combination of borate glasses with modifying oxides has been observed to have varying effects^11^. One important oxide modifier that enters the system is $\hbox {Li}_{2}$O, which does so by rupturing B-O-B bonds and causing bonding abnormalities. Ionic conductivity, thermal stability, transparency in the UV-NIR region and humidity resistance are all enhanced when $\hbox {Li}_{2}$O is combined with a borate network^12^. Lithium ions (Li^+^) form non-bridging oxygen species (NBOs) within borate glasses. The introduction of NBOs induces alterations in the characteristics of glass. Zinc-borate glasses demonstrate notable chemical resilience, rendering them suitable for application as soldering materials^13^. The characteristics of glasses containing noble metal ions or nanoparticles have been the subject of extensive research in the past decade, rendering them suitable for various optics, electronics and telecommunications applications. The controlled oxidation state of the metal ions and the size of these metal nanoparticles play a crucial role^14^. It has been discovered that adding copper (Cu) to borate glass improves its mechanical strength, stability and framework characteristics. Silver can be incorporated into the glassy matrix in two different ways: either as silver nanoparticles or as silver ions ($\hbox {Ag}^{+})$ depending on the concentration of silver dopant and the glass network^14^.

The objective of this study is to synthesize innovative amorphous borosilicate glass specimens that have been doped with newly developed copper nano-oxide and CuO/Ag nanocomposites. These samples will possess a crystalline phase and their analysis using XPS, XRD, SEM and HR-TEM techniques will be conducted individually. Furthermore, an investigation was conducted on the glass-doped nanocomposites created using ESR and UV analysis. The purpose of this study is to observe the alterations that took place in the nanocomposite following doping inside the glass matrix. Additionally, the gamma RdSg fields of the nanocomposites were examined. The LAc coefficient exhibited a considerable decrease as the content of CuO was increased. Among the synthesized CuB-1/CuAgB-X samples, the glass sample with the lowest CuO content exhibits the lowest HV, TV and MF values, indicating its superior gamma RdSg capabilities.

## Materials and methods

### Material

All metal salts and starting materials were of HPLC analytical grade or higher (Sigma Aldrich (USA)).

### Synthesis of CuO and Cu/Ag nanocomposites

#### General procedure

CuO and Ag/CuO nanocomposites were synthesized using sonication-solgel method^15–17^. 0.45 g of KOH (8.05 mmol) was mixed mechanically with 1 g copper (II) acetate monohydrate (5.01 mmol) and then added to PEG solution (300 mL DD (double distilled water), 0.60 g) under sonication for 20 min (12 cycles (2 min + 2 min pause)). Silver nitrate solution (50 mL) was added (over 8 min) to the mixture and continued sonication for 40 min. The mixture was put under stirring (900 rpm) at 85 $^{\circ }$C. The resulting solution was evaporated entirely before being crushed into granules. The resulting powders were calcined at 650 $^{\circ }$C for five hours in a furnace (air environment), crushed , washed with DD three times and dried

#### Starting materials

Cu-1: Silver nitrate solution (0.00 g), CuAg-2 [(CuO)_0.44_ (Ag)_0.56_]: Silver nitrate solution (1.10 g, 6.48 mmol), CuAg-3 [(CuO)_0.35_(Ag)_0.65_]: Silver nitrate solution (1.60 g, 9.42 mmol) or CuAg-4 [(CuO)_0.25_(Ag)_0.75_]: Silver nitrate solution (2.50 g, 14.72 mmol).

### Synthesis of glass

**Figure 1:**
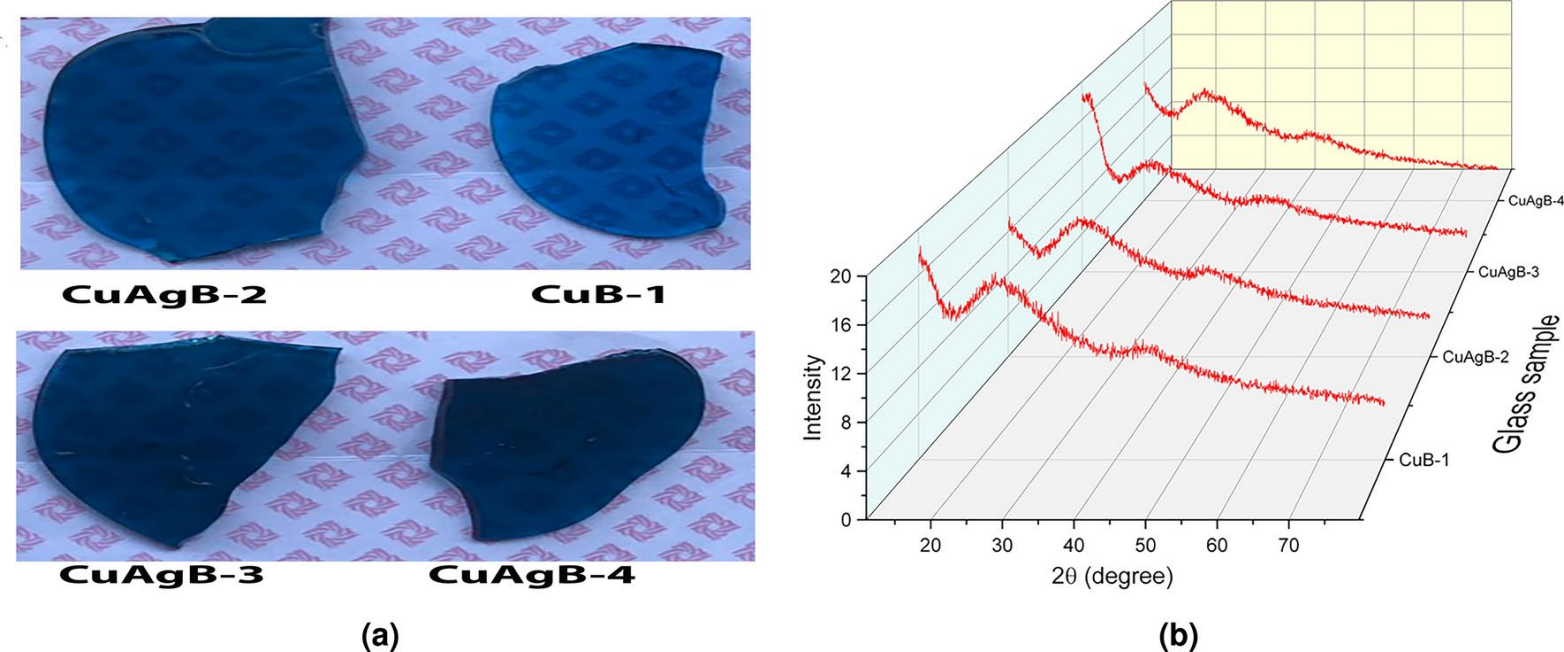
(**a**) Image of the prepared CuB-1/CuAgB-X samples, (**b**) XRD patterns of the prepared glass samples.

The samples were produced by melting H_3_BO_3_ (18.19 g, 233.73 mmol), Li_2_CO_3_ (3.84 g, 51.91 mmol), SiO_2_ (1.26 g, 21.02 mmol), ZnO (1.71 g, 21.01 mmol)) and 2.50 g of the previously prepared nano-oxide/nanocomposites (CuB-1: Cu-1, CuAgB-2: CuAg-2, CuAgB-3: CuAg-3 and CuAgB-4: CuAg-4) in a furnace at a temperature of 1000 $^{\circ }$C in a 50 mL porcelain crucible. After 1 hour, approximately 25 g of glass sample was obtained. The molten phase of the samples was solidified by being cooled on a stainless-steel mold. Subsequently, it was promptly transferred to another pre-heated furnace to a temperature of 300 $^{\circ }$C. The samples were then allowed to gradually cool to room temperature overnight, with a cooling rate of approximately 12 $^{\circ }$C per minute. Prepared samples underwent a process of polishing/cutting to obtain 0.75 mm in thickness. Images of the prepared samples are shown in Fig. 1a.

### Experimental techniques

The XPS analysis was conducted using “monochromatic X-ray Al K-alpha radiation” with an energy range of − 10 to 1350 eV. The spot size used was 400 micrometers and the pressure during the analysis was maintained at 10^-9^ mbar. Sonifier®SFX550 with 550 W of power at 20 kHz for high-volume processing. The XRD patterns were obtained from Brucker D8 discovered using Cu-radiation ( $\lambda$ = 1.542 A). The average size of the crystallites was calculated by Debye–Scherrer equation. The morphology was analyzed using a Philips SEM instrument, model QUANTA FEG 250, manufactured in the USA. The powder’s morphology, size and surface structure were analyzed using a (TEM) transmission electron microscope. Sonifier®SFX550 with 550 watts of power at 20 kHz for high-volume processing. ESR spectra were obtained utilizing (ER 4102) model of the “ESR-EMX/Burker” spectrometer operating at 9.5 GHz of a x-band frequency with range started from 2800 to 4100 Gauss of the magnetic field. The ESR settings were set to a microwave power of fixed value of 0.796 mV settled up by the conversion time of 20.48 ms

### Density calculations

Density ($\rho$) serves as a crucial physical metric in understanding the characteristics of glass samples, as it quantifies the relationship between their masses and volumes. The density was determined using Archimedes’ Principle, as expressed in Equation .

1 $$\begin{aligned} \rho = \frac{W_{air}}{W_{air}-W_{xylene}}. \end{aligned}$$

Whereas, $\rho$ is the density of the glass and $\hbox {W}_{air}$ and $\hbox {W}_{xylene}$ are the weight of the specimen in air and deepen in xylene, respectively , see Table 1.

**Table 1:** The molfs and densities of the prepared CuAgB-X samples.

Sample ID	Oxides composition, (mol%)						Density
	$$\hbox {B}_{2}$$$$\hbox {O}_{3}$$	$$\hbox {Li}_{2}$$O	$$\hbox {SiO}_{2}$$	ZnO	CuO	Ag	(g $$\hbox {cm}^{3}$$)
CuB-1	48.25	21.43	8.68	8.68	12.97	0.00	2.34
CuAgB-2	49.19	21.85	8.85	8.85	5.77	5.50	2.37
CuAgB-3	49.34	21.92	8.87	8.87	4.61	6.38	2.39
CuAgB-4	49.50	21.99	8.90	8.90	3.38	7.32	2.47

### Gamma-rays attenuation investigation

#### MCNP simulation

**Figure 2:**
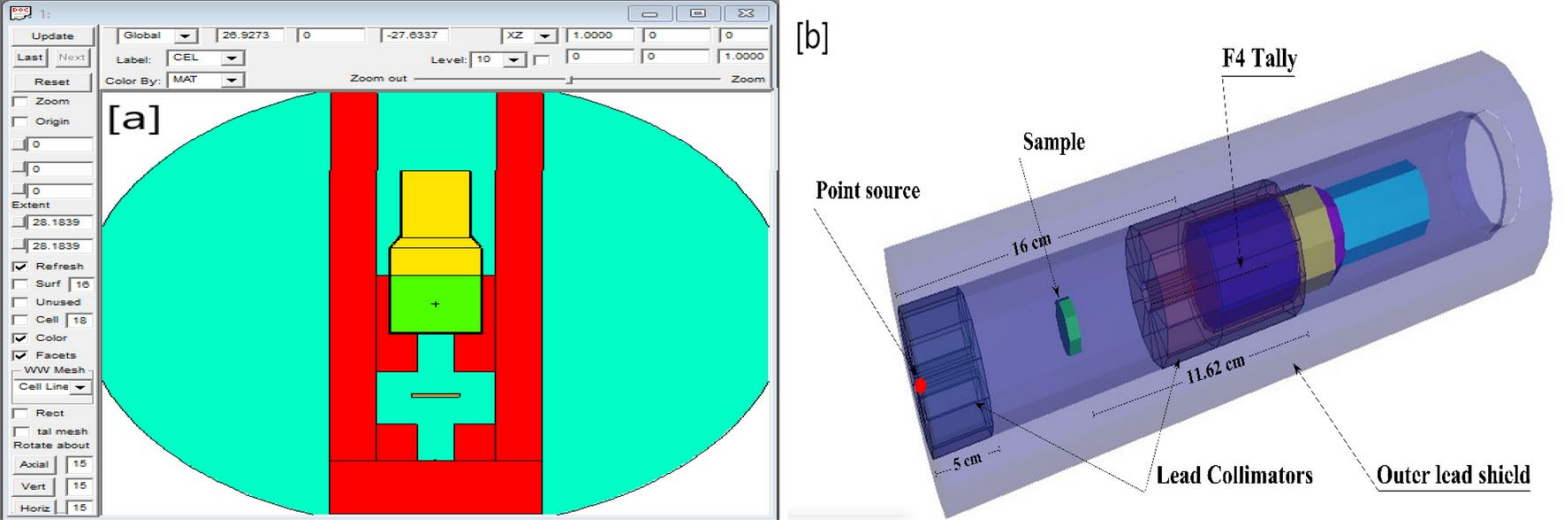
The created simulation system in (**a**) 2D and (**b**) 3D views for the synthetic CuB-1/CuAgB-X samples.

The gamma radiation simulations of the synthetic CuB-1 and CuAgB-X glasses were achieved via the Monte-Carlo simulation code-5 (Mc) with a mono-energy $\gamma$-point source in the ${\gamma }$E of ( 0.15e-1 to 15 MeV). It stimulates the transit of $\gamma$-rays while considering the physics of interaction with matter (photo-electric (PEE), compton (COM) and pair production (PaP))^18^ through the E-N-D-F/B-V-I-8 nuclear database^19^. The input files for the Mc simulation depend on the detailed structure of the input file code, which consists of multiple cards (cell, surface, material, tally, etc.) including the experimental setup (e.g., detector dimensions, sample geometry, source height, chemical composition, etc.)^20^. The $\gamma$-ray shielding set-up was described in the TEXT file in many cell cards (e.g., the $\gamma$ source, two collimators, sample and detector)^21^. The F4:P tally card was used to determine the track length of the incident $\gamma$-photons^22^. The CuB-1/CuAgB-X samples were created in cylinder geometry and their composition and densities were created in the material card of the input file. The number of particles emitted from the $\gamma$-source (NPS) was created to be more than 9999999 particles per input file to reduce the random statistical errors to be below 2$\%$. Figure 2a,b represents the dimensions of the radiation-simulated system used for the investigated glasses.

The Lambert–Beer’s law, calculates the LAC, $\mu$ as follows^23,24^:

2 $$\begin{aligned} \mu&=-\frac{1}{x}ln(\frac{I}{I_{o}}). \end{aligned}$$

Where, I and $\hbox {I}_{o}$ are the intensity of air and transferred $\gamma$-rays through the material, respectively, $\chi$ is the sample’s thickness. The half-value layer (HV) and the tenth-value layer (TV) parameters calculate the values needed to reduce the incident radiation to half or a tenth of its original value. The HV and the TV can be calculated using Eqs. (3) and (4)^25–27^:

3 $$\begin{aligned} HV&=\frac{ln2}{\mu }, \end{aligned}$$

4 $$\begin{aligned} TV&=\frac{ln10}{\mu }. \end{aligned}$$

The mean free path (MF) is the shielding parameter that describes the distance that $\gamma$-ray photons travel inside the shielding material between two successive collisions, which is determined through the following equation^25,26^:

5 $$\begin{aligned} MF&=\frac{1}{\mu }. \end{aligned}$$

#### Phy-X/PSD software

Online PhyX/PSD software (PhyX) calculates shielding/attenuation factors for material compositions^28^. To analyze the CuB-1/CuAgB-X glasses, Most RdSg characteristics were selected as output data^29^. The relative differences ($\Omega$, $\%$) were estimated by comparing the data received from PhyX and Mc as follows:

6 $$\begin{aligned} \Omega , (\%) =\frac{MCNP-PhyX}{MCNP} \times 100. \end{aligned}$$

## Results and discussion

### XPS

**Figure 3:**
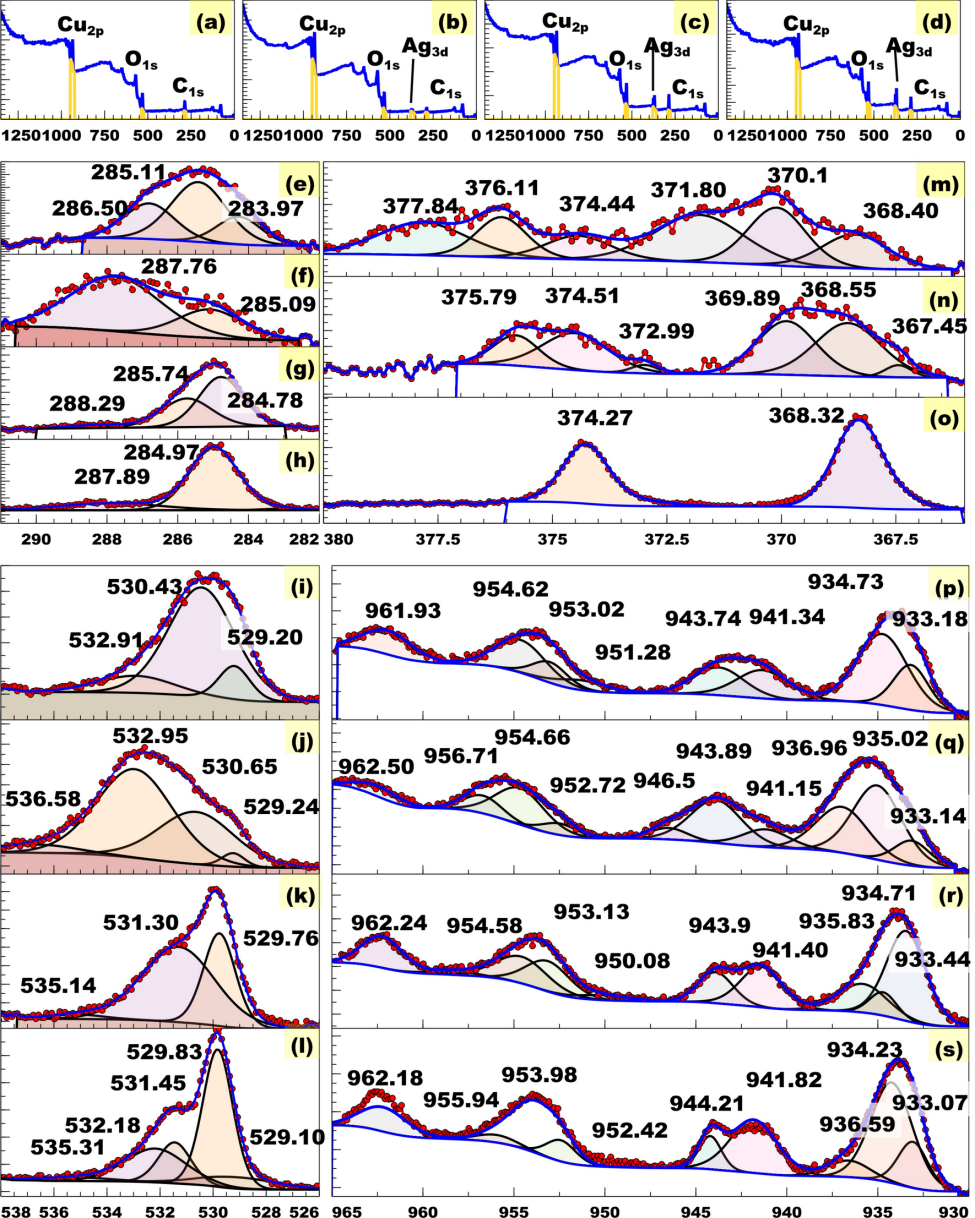
Survey scan spectra of (**a**) Cu-1, (**b**) CuAg-2, (**c**) CuAg-3 and (**d**) CuAg-4, HRs C-1s XPS spectra of (**e**) Cu-1, (**f**) CuAg-2, (g) CuAg-3 and (**h**) CuAg-4, HRs O-1s XPS spectra of (**i**) Cu-1, (**j**) CuAg-2, (**k**) CuAg-3 and (**l**) CuAg-4, HRs Ag-3d XPS spectra of (**m**) CuAg-2, (**n**) CuAg-3 and (**o**) CuAg-4 and HRs Cu-2p XPS spectra of (**p**) Cu-1, (**q**) CuAg-2, (**r**) CuAg-3 and (**s**) CuAg-4.

Figure 3 shows the XPS spectra of nano-materials. All survey spectra (Fig. 3a–d) show the presence of copper (Cu-2p, 934.85–935.45 eV) and oxygen (O-1s, 531.26–532.16 eV) and all except that of Cu-1 indicate the presence of silver (Ag-3d, 369.10–370.12 eV)(see Fig. 3b–d). The presence of C (C-1s, 285.93–287.54 eV) may be due to the adsorption on the sample’s surface, as a residue, or during sample preparation. The HRs C-1s XPS of the nano-materials (Fig. 3e–h) show multiple peaks in the 283.97–287.89 eV region. The spectrum of Cu-1 shows peaks at 283.97 and 286.50 which may be attributed to C=C and $\alpha$-carbon, respectively^30,31^. The spectra of CuAg-3 and CuAg-4 show peaks in the 284.78–284.90 eV region which may be attributed to sp^2^-bonded carbon (C=C), while that of CuAg-2 and CuAg-4 show peaks in the 287.76–287.89 eV region which may be attributed to carbonyl group^32,33^. The spectra of Cu-1 and CuAg-2 show peaks in the 285.09–285.11 eV region which can be attributed to C–C (sp^3^ carbon) while the spectrum of CuAg-3 shows peak at 285.74 eV which can be attributed to C–OH^34,35^. The HRs O-1s XPS spectra (3i–l) of all nano-materials show peaks in the 529.10–529.83 eV region attributed to metal oxygen^36,37^. The spectra of Cu-1 and CuAg-2 show peaks in the 530.43–530.65 eV region which can be attributed to surface adsorbed oxygen ($\hbox {O}_{\beta }$)^38^. The spectra of CuAg-3 and CuAg-4 show peaks in the 531.30–531.45 eV and 535.14–535.31 eV regions which can be attributed to O=C and $\hbox {O}_{\gamma }$ associated with carbonate species, respectively^30,38^. The spectra of Cu-1 and CuAg-2 show peaks in the 532.91–532.95 eV region which can be attributed to chemisorbed oxygen while the peak at 532.18 eV in the spectrum of CuAg-4 was attributed to superoxide species which result from the reaction of O_2_ and oxygen vacancies^38,39^. All of theses spectrum, except Cu-1 shows peaks in 535.14-536.58 eV which can be attributed to gas phase water^40^. The HRs Ag-3d XPS spectra of nanocomposites is shown in Fig. 3m–o. This is supported by the two spin-orbit doublets (3d_5/2_ and 3d_3/2_) appearing in 367.45–368.55 and 372.99–374.51 eV, respectively^41,42^. All of these spectra show two spin-orbit doublets in the 368.32–368.55 and 374.27–374.51 eV regions which can be attributed to 3d_5/2_ and 3d_3/2_, respectively in Ag^0^ while the extra spin-orbit doublets shown by CuAg-3 at 367.45 and 372.99 eV can be attributed to 3d_5/2_ and 3d_3/2_, respectively in Ag^+^. The ratio of oxidized silver can be calculated from Eq. (7)

7 $$\begin{aligned} \% [Ag^{+}]&= \frac{\sum Ag^{+} \hspace{2pt} PAR \hspace{2pt} ( 3d_{5/2} + 3d_{3/2}) \hspace{5pt}x \hspace{2pt}100 }{\sum Ag^{+} \hspace{2pt} PAR \hspace{2pt} ( 3d_{5/2} + 3d_{3/2}) + \sum Ag \hspace{2pt} metal \hspace{2pt} PAR \hspace{2pt} ( 3d_{5/2} + 3d_{3/2})}. \end{aligned}$$

The result shows, only 8.38% was oxidized at the surface of the sample, which indicate a high stability of the prepared nano-material. The spectrum of CuAg-2 and CuAg-2 shows doublets in the regions 369.89–371.80 eV and 375.79–377–84 eV. These peaks with high binding energy are assigned to Ag satellite peaks, which result in asymmetric line shapes^43^. The HRs Cu-2p XPS spectra are shown in Fig. 3p–s. All spectra show two spin-orbit doublets in the (9.33.07–933.44, and 952.72–953.98 eV) and (934.23–935.02 and 954.58–955.94 eV) regions, which can be attributed to 2p_3/2_ and 2p_1/2_ in $\hbox {Cu}^{+}$ and $\hbox {Cu}^{2+}$, respectively^44,45^. The absence of any peaks below 933 eV indicates that Cu^0^ was absent^46–48^. The ratio of $\hbox {Cu}^{2+}$ can be calculated using Eq. (8).

8 $$\begin{aligned} \% [Cu^{2+}]&= \frac{\sum Cu^{2+} \hspace{2pt} PAR \hspace{2pt} ( 2p_{3/2} + 2p_{1/2}) \hspace{2pt} x \hspace{2pt}100 }{\sum Cu^{2+} \hspace{2pt} PAR \hspace{2pt} ( 2p_{3/2} + 2p_{1/2}) + \sum Cu^{+} \hspace{2pt} PAR \hspace{2pt} ( 2p_{3/2} + 2p_{1/2})}. \end{aligned}$$

The values of % $\hbox {Cu}^{2+}$ were 69.58, 80.64, 29.45 and 57.69 for Cu-1, CuAg-2, CuAg-3 and CuAg-4, respectively. These support the presence of CuO by many peaks including: characteristic $\hbox {Cu}^{2+}$ peaks in 935.83–936.96 eV region (all except Cu-1), multible peaks associate with CuO in 941.15–952.72 eV region and at 956.71 (CuAg-3 only) and the unfilled Cu 3$\hbox {d}^{9}$ shell in 961.93–962.50 eV region^49–51^.

### XRD characterization

**Figure 4:**
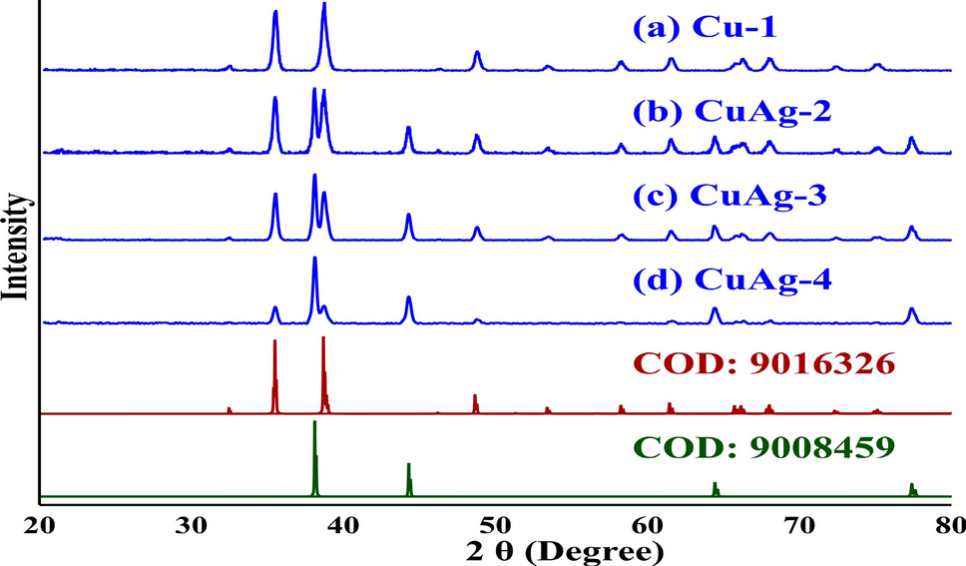
XRD spectra of (**a**) Cu-1, (**b**) CuAg-2, (**c**) CuAg-3, (**d**) CuAg-4, COD: 9016326 and COD: 9008459.

CuO and Ag/CuO nanocomposite gave sharp swell-defined XRD peaks indicating good crystallinity patterns, Fig. 4a–d. The XRD patterns of Cu-1, CuAg-2, CuAg-3 and CuAg-4 were represented in Fig. 4a–d and matched by XRD reference codes COD: 9016326 for CuO, while that of CuAg-2, CuAg-3 and CuAg-4 were also matched by XRD reference codes COD: 9008459 for Ag. The XRD patterns of all samples showed diffraction peaks for tenorite with space group C 1 c 1 (9), see Table 2. The XRD patterns of CuAg-2 and CuAg-3 showed four very similar values of 2$\theta$ while these peaks were slightly shifted in case of CuAg-4, see Table 2. These peaks may be indexed to (1 1 1), (2 0 0), (2 2 0) and (3 1 1) for Ag with space group F m -3 m (225). In the case CuO, the average particle size was calculated using the most three intense peaks while in the case of Ag all peaks were used in the calculations. CuO particles sizes were 29.32, 31.58, 31.53 and 35.80 nm for Cu-1, CuAg-2, CuAg-3 and CuAg-4, respectively, while that of Ag were 45.04 (CuAg-2), 45.95 (CuAg-3) and 35.13 nm (CuAg-4). Table 2 shows the most important 2$\theta$s and their hkl values. The peaks in 38.69$^{\circ }$–38.72$^{\circ }$ and 35.53$^{\circ }$–35.56$^{\circ }$ region of CuO and the peak in 38.08$^{\circ }$–38.10$^{\circ }$ region of Ag may be used to indicate the CuO:Ag ration through samples from Cu-1 to CuAg-4, see Fig. 4. In Cu-1 the peak around 38.10 was not found which support the absence of silver in this sample. As the concentration of silver increase compared to that of CuO, the intensity of CuO peaks also decreases comparing to that of Ag (Fig. 4). Structural parameters and the calculated cell parameters of all phases are given in Table 3.

In another hand; XRD analysis has been measured for all glass samples doped with the previously prepared nanocomposite. As given in Fig. 1b all samples showing the amorphous nature of the glass samples that prove the excellent distribution of the nanocomposites inside the glassy network without any coagulation. The absence of peaks in the spectra for all glass samples means the disappearance of the crystalline phase.

**Table 2:** The most important 2$\theta$s, their hkl values and the calculated cell parameters.

CuO	Cu-1	CuAg-2	CuAg-3	CuAg-4	Ag	CuAg-2	CuAg-3	CuAg-4
h k l	Angle [$^{\circ }2\theta$]				h k l	Angle [$^{\circ }2\theta$]		
1 1 0	32.5	32.5	32.49	32.53	1 1 1	38.1	38.1	38.08
0 0 2	35.43	35.43	35.46	35.41	2 0 0	44.28	44.28	44.26
1 1 − 1	35.53	35.54	35.53	35.56	2 2 0	64.41	64.41	64.37
1 1 1	38.69	38.69	38.7	38.72	3 1 1	77.36	77.36	77.31
2 0 0	38.94	38.94	38.94	38.89				
1 1 − 2	46.25	46.26	46.26	46.26				
2 0 − 2	48.77	48.77	48.77	48.71				
1 1 2	51.31	51.32	51.34	51.33				
0 2 0	53.42	53.43	53.41	53.54				
0 2 − 1	56.66	56.67	56.65	56.77				
2 0 2	58.25	58.25	58.28	58.21				
1 1 − 3	61.53	61.54	61.57	61.53				
0 2 − 2	65.74	65.76	65.76	65.84				
3 1 − 1	66.26	66.27	66.25	66.21				
3 1 0	66.49	66.49	66.47	66.44				
1 1 3	67.85	67.85	67.91	67.85				
2 2 0	68.05	68.07	68.04	68.13				
2 2 − 1	68.86	68.87	68.84	68.93				
3 1 − 2	71.73	71.73	71.72	71.67				
3 1 1	72.37	72.37	72.37	72.32				
2 2 1	72.89	72.9	72.89	72.96				
0 0 4	74.96	74.96	75.05	74.93				
2 2 − 2	75.22	75.24	75.22	75.28				
0 2 − 3	79.67	79.68	79.7	79.75				

### SEM, HRTEM and particle diameters distributions

**Figure 5:**
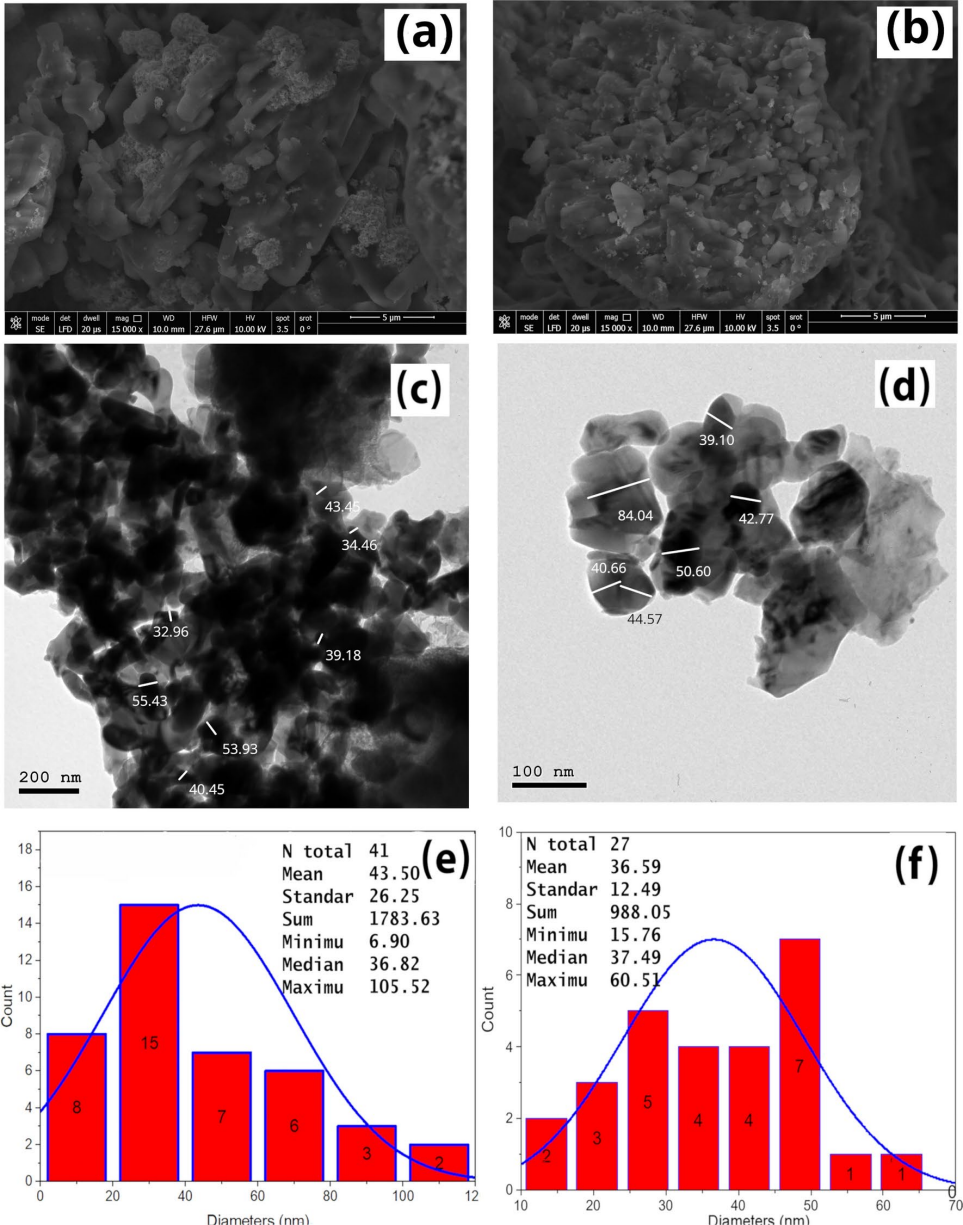
SEM of (**a**) CuAg-3 and (**b**) CuAg-2, HRTEM of (**c**) CuAg-4 and (**d**) CuAg-2 and particle diameters distributions of (**e**) CuAg-3 and (**f**) CuAg-4.

The microstructure of the developed CuAg-3 and CuAg-2 samples was analyzed using SEM images with large field detector LFD diffraction. Figure 5a,b reveal that the particles are compact, irregularly shaped and interspersed with rough, porous surfaces. The particle sizes vary widely, from small, nearly spherical forms to larger, more elongated shapes. The particles also tend to clump together, forming aggregates in various shapes, some resembling a mushroom-like structure. Additionally, two distinct color contrasts are visible, likely due to the differences in electron absorption between the $\hbox {Cu}^{2+}$ and Ag metals. In Fig. 5a, the CuAg-3 sample, which contains more Ag than the CuAg-2 sample shown in Fig. 5b, displays Ag particles as spherical, dense dots with uniform size and distribution, decorated relatively smooth CuO particles. Figure 5c,d present the TEM micrographs of the CuAg-3 and CuAg-4 nanocomposites. The particles exhibit irregular shapes and a relatively wide size distribution, ranging from 32 to 85 nm. Additionally, two distinct contrast areas are visible: a bright area corresponding to CuO nanoparticles, which appear as nano-sheets and a dark area representing Ag nanoparticles. The contrast is attributed to the difference in electron absorption^52^, with $\hbox {Ag}^{0}$ metal (the density: $\sim$10.50 g/$\hbox {cm}^{3}$) being denser than CuO ($\sim$ 6.50 g/$\hbox {cm}^{3}$). Moreover, some aggregation is observed, likely due to the high surface energy and collision frequency of the nanoparticles, as well as dipole-dipole interactions^52^. Notably, the mean particle sizes were 43.50 nm for Ag and 36.59 nm for CuO, which are consistent with the values derived from the most intense XRD peaks: CuAg-3 (CuO ($\sim$ 31.53 nm) and Ag ($\sim$ 45.95 nm)) and CuAg-4 (CuO ($\sim$ 35.80 nm) and Ag ($\sim$ 35.13 nm)). Figure 5: e and f show the particle size distribution of CuAg-3 and CuAg-4, respectively. The mean values were 43.50 and 36.59 nm, respectively. These values were close to that calculated in using the most intense XRD peak (CuAg-3: CuO (31.53 nm); Ag (45.95 nm) and CuAg-4: CuO (35.80 nm), Ag (35.13 nm)).

**Table 3:** Atomic and structural parameters.

	Cu-1	CuAg-2		CuAg-3		CuAg-4	
	CuO	CuO	Ag	CuO	Ag	CuO	Ag
a (COD value)	4.68 (4.69)	4.69 (4.69)	4.09 (4.09)	4.69 (4.69)	4.09 (4.09)	4.69 (4.69)	4.09 (4.09)
b (COD value)	3.43 (3.43)	3.43 (3.43)		3.43 (3.43)		3.42 (3.43)	
c (COD value)	5.13 (5.14)	5.13 (5.14)		5.13 (5.14)		5.14 (5.14)	
$\alpha = \gamma$	90.00$^{\circ }$(90.00$^{\circ }$)						
$\beta$	99.43$^{\circ }$ (99.55$^{\circ }$)	99.42$^{\circ }$ (99.55$^{\circ }$)	90.00$^{\circ }$ (90.00$^{\circ }$)	99.45$^{\circ }$ (99.55$^{\circ }$)	90.00$^{\circ }$ (90.00$^{\circ }$)	99.45$^{\circ }$ (99.55$^{\circ }$)	90.00$^{\circ }$ (90.00$^{\circ }$)
2 $\theta$	38.69	38.69	38.10	38.70	38.10	38.72	38.08
Microstrain ($\epsilon$ × 10^−3^)	3.48	3.48	2.27	3.23	29.82	3.73	3.03
Specific surface area (S m^2^ g^-1^)	31.12	30.90	12.37	28.82	162.25	33.26	16.50
Delayed diffractions	2.41	2.41	2.48	2.41	2.48	2.41	2.49
Atomic scattering polarization factor	0.80	0.80	0.81	0.80	0.81	0.80	0.81
Lorentz polarization factor	15.54	15.54	16.08	15.53	16.08	15.52	16.10

### ESR of glass samples doped nanocomposites

**Figure 6:**
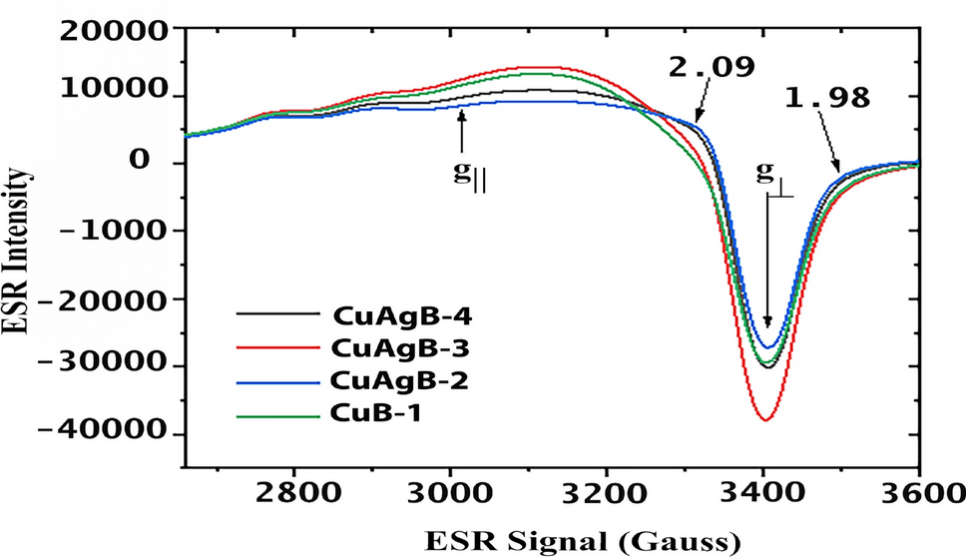
ESR spectra of glass samples

ESR can help get valuable information about silver and copper, where Ag and Cu can be paramagnetic ions and their aggregates inside the network. ESR spectra of all nanoparticles with difrent ratios between silver and copper (Table 1) have been depicted in Figure 6. Sample CuB-1 free of Ag was represented to compare with other samples doped with different ratios of Ag/CuO. ESR spectra were not very sensitive to silver dopants since all the resonance ESR signals were indications for only Cu dopants with poorly changed after silver dopants as weak signals have been recorded related to Ag paramagnetic species that may be due to the effect of aggressive heating on the samples during melting to create Ag^+^ or Ag^2+^^53^ in agglutinated or isolated species from Ag^0^^53^. The ESR spectrum of doped glass specimens reveals that the signals for CuAgB-4 and CuAgB-2 exhibit a similar shape, albeit with minor variations in intensity. Conversely, the shape of the signals for samples CuAgB-3 and CuB-1, which have different concentrations of Cu/Ag, display tiny alterations in the form of a g-value of 2.09 and others at 1.98. The spectra presented in this study exhibit a remarkable similarity to the spectra obtained by previous studies. In the current spectra, three weak parallel components were observed in the lower field region, while a fourth component was found to overlap with a perpendicular component. By many authors^54^ using ESR spectroscopy, the incorporation of Cu ions into a glass network as a function of the copper content was investigated. Copper ions are probably present as Cu^+^ and Cu^2+^ in the matrix of these glasses; however, only Cu^2+^ by a 3d^9^ electronic structure and a ground state of 2 D_3/2_ can be identified by ESR spectroscopy, which is used at ambient temperature. The electron paramagnetic resonance (EPR) spectra consist of a mixture of two bands. The presence of isolated Cu^2+^ ions exhibiting axial symmetry manifests a hfs, characterized by a g$\parallel$ value of 2.4. The presence of another broad signal with a g$\perp$-value of 2.08, previously mentioned in other studies, may be attributed to the interaction among neighboring Cu^2+^ ions^54^. Generally, these spectra’ hfs exhibits poor resolution, with only a limited number of cases delineating the hfs features^55,56^. The ESR spectrum illustrates a combination of absorption signals from both isolated Cu^2+^ ions (found at sites with an axial environment) and coupled Cu^2+^ ions, as previously indicated^56^. The initial signal has a poorly developed hfs for the parallel band and remains unclear for the perpendicular band. According to the research conducted by Ciorcas et al.^56^ strong ligand field fluctuations within the glassy matrix may account for the anisotropic hfs, resulting in line broadening. The presence of anisotropy leads to the broadening of spectral lines, which can be attributed to the strengthening of long-range interactions as the concentration of CuO increases. Therefore, based on the findings of Dehelean et al.^57^ it may be inferred that the symmetry that exists between the isolated ions as well as the interacting ions, which constitute a cluster of mutually interacting ions, is either similar or there are specific ions that exist in isolation without being part of any cluster. In our studied samples, it has been determined that g$\parallel$ > g$\perp$ . This finding suggests that Cu^2+^ ions were found as tetragonal coordinates that were distorted octahedral sites^58^. The obtained result indicates that the Cu^2+^ ions in the current models are coordinated by six ligands, forming a CuO_6_ chromophore. The Cu^2+^ ions form octahedral structures that are elongated along the z-axis. This occurrence signifies that the ground state of Cu^2+^ ions correspond to the dx_2_-y_2_ orbital, namely the 2B_1g_ state. The copper nuclei forms ^63^Cu and ^65^Cu exhibit an electronic spin (S) of 1/2 and an overall nuclear spin (I) of 3/2 in the form of Cu^2+^ ions^59^. Furthermore, the singular and extensive absorption observed at g $\approx$ 2.1, indicative of connected Cu^2+^ pairs (Cu–O–Cu) and clustered Cu^2+^ ions, exhibits increased symmetry. It should be noted that the absence of detected ESR absorptions resulting from the presence of Ag ions does not necessarily indicate their lack in the samples under investigation. In this particular scenario, the concentration of Ag ions is comparatively lower than the threshold detected by ESR spectroscopy. Alternatively, the absorptions attributed to these ions exhibit a notably weak intensity, overlapped by the more significant absorptions generated by the Cu^2+^ ions^60^. The silver ions create places where electrons become trapped when establishing Ag^0^ atoms. Since Ag^0^ atoms are inherently unstable, they interact with Ag^+^ ions to form more stable $\hbox {Ag}^{x+}_{y}$ clusters. Changes to the Ag species in the glass can happen when the glass is melted. The high mobility of silver and interaction between the Ag^0^ and Ag^+^ then facilitates the construction of silver clusters^53,61,62^. Some silver species are paramagnetic, including Ag^0^ (4d^10^ 5s^1^), Ag^2+^ (4d^9^) and several silver nanoclusters^62,63^. From the ESR spectrum; Ag^2+^ results from signals appear at g 2.09. Concerning the clusters of paramagnetic silver, $\hbox {Ag}^{2+}_{3}$ it is suggested they generate weak absorption at g 1.98. According to ESR spectroscopy, converting Ag^+^ ions to Ag^0^ atoms does not occur in the studied samples. Since no quantities of Ag^0^ were detected (at g-value=2.2), the possibility of $\hbox {Ag}^{+}_{2}$ clusters (at g-value 1.99) is also negligible. This confirms our hypothesis that samples doped with Ag/CuO only contain Ag^2+^ ions and, $\hbox {Ag}^{+}_{2}$ clusters without additional silver ionic Ag^+^ or $\hbox {Ag}^{+}_{2}$ agglutinated forms^14^. It can be concluded that; samples CuB-1 with Cu only give ESR spectra similar to CuAgB-3 which may be due to the presence of Ag ions in this sample as Ag^+^ or $\hbox {Ag}^{+}_{2}$ that do not give any response in ESR spectra while in the case of samples CuAgB-4 and CuAgB-2, the silver ions exist as $\hbox {Ag}^{+}_{2}$ and $\hbox {Ag}^{2+}_{3}$.

### Physical study of the prepared glass

**Figure 7:**
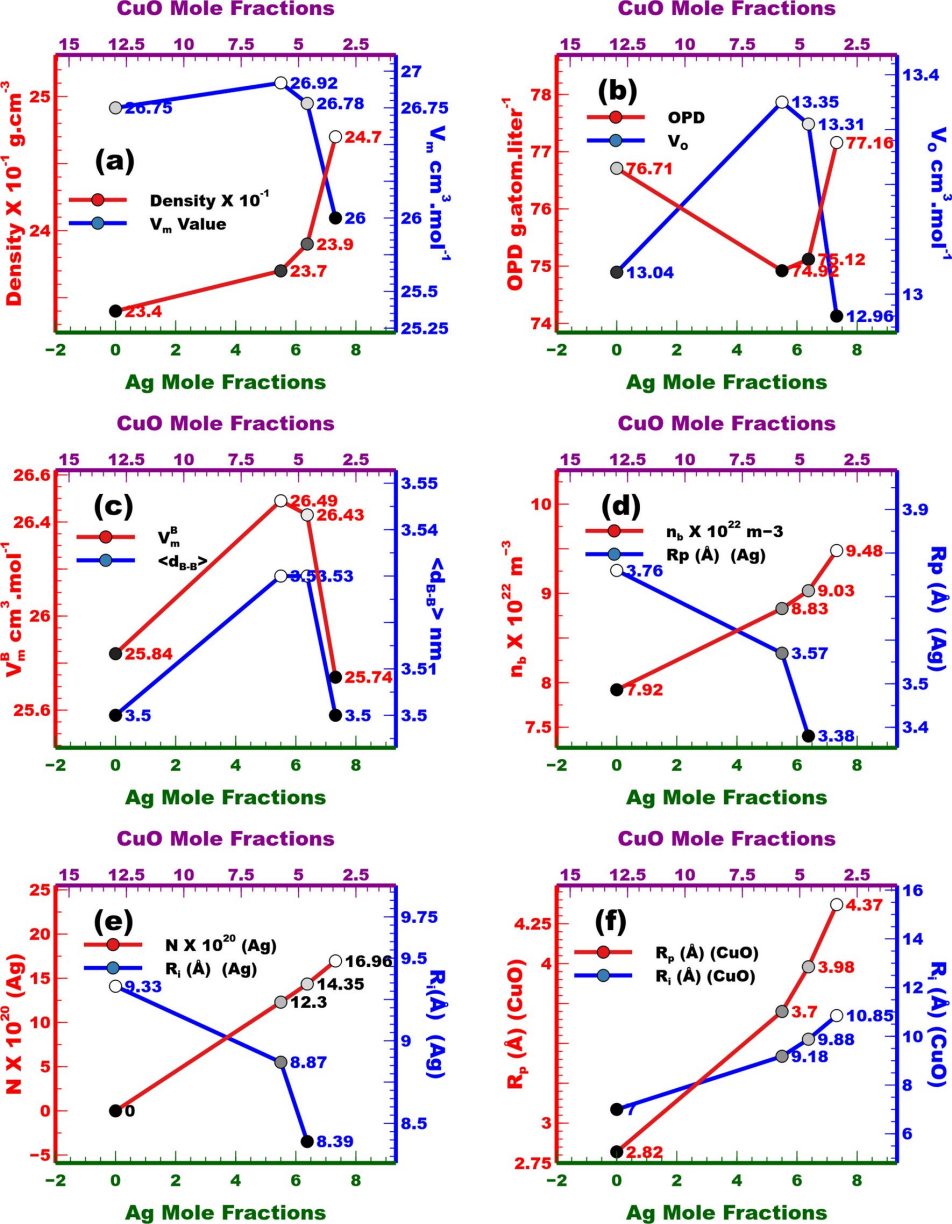
Effect of changing CuO/Ag mole fraction on (**a**) density and $\hbox {V}_{m}$, (**b**) OPD and $\hbox {V}_{o}$, (**c**) $\hbox {V}_{m}^{B}$ and $\hbox {d}_{B-B}$ , (**d**) $\hbox {n}_{b}$ and R*p*(Å) (Ag) and (**e**) N (Ag) and $\hbox {R}_{i}$(Å) (Ag) and (**f**) R*p*(Å) (CuO) and $\hbox {R}_{i}$(Å) (CuO).

Changing metal oxide and/or their ratios have a high effect on the density, which in turn have a high impact on the physical properties of the produced glass^64,65^. In current study, we investigate the effect of increasing the molf of silver metal along with decreasing the molf of CuO in a borosilicate glass. Tables 1, 4 and Fig. 7a, show the relation between densities and Ag molf. Densities increased from the 23.40 g $$\hbox {cm}^{-3}$$ (0 % Ag) to 24.70 g $$\hbox {cm}^{-3}$$ (7.32 % Ag). These results depend mainly on the high density of Ag metal (10.50 g $$\hbox {cm}^{-3}$$) compared with other metal salts ($$\hbox {B}_{2}$$$$\hbox {O}_{3}$$ (2.46 g $$\hbox {cm}^{-3}$$), $$\hbox {Li}_{2}$$O (2.01 g $$\hbox {cm}^{-3}$$), $$\hbox {SiO}_{2}$$ (2.32 g $$\hbox {cm}^{-3}$$), ZnO (5.61 g $$\hbox {cm}^{-3}$$) and CuO (6.31 g $$\hbox {cm}^{-3}$$)). The irregularity noticed in this figure may be due to a structural modification caused by the difference in size and $$\hbox {C}_{no}$$ between $$\hbox {Cu}^{2+}$$ and $$\hbox {silver}^{0}$$. Equation (9) to (12) were used to calculate the molar volume ($$\hbox {V}_{m}$$), $$\hbox {V}_{o}$$, $$\hbox {V}_{m}^{B}$$ and $$\hbox {d}_{B-B}$$ of glass samples, respectively, where, $$\hbox {x}_i$$ is the molf and $$\hbox {M}_{wt-i}$$ is its molar mass fraction.

9 $$\begin{aligned} V_{m-i}&= \frac{\Sigma x_i M_{wt-i}}{\rho _{i}}, \end{aligned}$$

10 $$\begin{aligned} V_{o}&= \frac{V_{m}}{\sum x_{i} .n_{i}}, \end{aligned}$$

11 $$\begin{aligned} V_{m}^{B}&= \frac{V_{m}}{2(1-X_{B})}, \end{aligned}$$

12 $$\begin{aligned} {<}d_{B-B}{>}&= (\frac{V_{m}^{B} }{2(1-X_{B})}) ^ \frac{1}{3}. \end{aligned}$$

Generally, $\hbox {V}_{m}$ decreases with the increase in density. At the beginning, $\hbox {V}_{m}$ showed an abnormal behavior (Table 4 and Fig. 7a), it increased slightly from 26.75 (CuB-1, 0.00 Ag) to 26.78 (CuAgB-3, 6.38 Ag) indicating an expansion of glass structural network occurring with the addition of silver (larger in size), which is also supported by the increase in $\hbox {V}_{o}$ value (Fig. 7b). Further increase in silver molf led to a decrease in both $\hbox {V}_{o}$ and $\hbox {V}_{m}$ values due to the higher $\hbox {C}_{no}$ of silver (12 (fcc)) compared to $\hbox {Cu}^{2}$ (4). This may led to a compression in glass structural network. $\langle \textrm{d}_{B-B}\rangle$ and $\hbox {V}_{m}^{B}$ (Table 4 and Fig. 7:c) followed the exact pattern as $\hbox {V}_{o}$ and $\hbox {V}_{m}$. OPD was calculated using Eq. (13) the number of oxygen’s in the glasses (c), the density ($\rho$) and sum of molar mass fraction.

13 $$\begin{aligned} OPD&= \frac{c.\rho .10^{3}}{M_{wt}}. \end{aligned}$$

Other than density, OPD have a direct impact on most glass physical properties. Table 4 and Fig. 7: b show the OPD values. The difference between initial value (Cu-1, 76.71) and final value (CuAgB-4, 77.16) is very small. The increase in density is usually occupied with an increase in OPD value, but in this case other factors, including oxidation number (Ag: c = 0, $\hbox {Cu}^{2}$: c = 2), $\hbox {C}_{no}$ (Ag: 12 (fcc), $\hbox {Cu}^{2}$: 4) and are playing important roles on the determination of OPD value. The values of OPD decreased from 76.71 (Cu-1) to 74.92 (CuAgB-2) then increase reaching maximum value of 77.16 at CuAgB-4. Other physical parameters were calculated using Eqs. (11)–(17).

14 $$\begin{aligned} N&= \frac{X_{Cu}.\rho .Na }{M_{w}}, \end{aligned}$$

15 $$\begin{aligned} R_{p}&= \frac{1 }{2} ( \frac{\pi }{6N})^ \frac{1 }{3}, \end{aligned}$$

16 $$\begin{aligned} R_{i}&= ( \frac{1}{N})^ \frac{1 }{3} \end{aligned}$$

17 $$\begin{aligned} n_{b}&= \frac{Na.\sum x_{i} .n_{f} }{V_{m}}. \end{aligned}$$

The value of N, $\hbox {R}_{p}$ and $\hbox {R}_{i}$ were calculated for CuO and Ag molfs (Table 4 and Figure 7d–f). Theses results indicated an increase of N (Ag) values from 0.00 (CuB-1, no Ag) to 16.96 (CuAgB-4, max Ag molf) and a decrease in N (CuO) values from 29.20 (CuB-1, max CuO molf) to 16.96 (CuAgB-4, min Ag molf) which come with agrement with the synthsis procedure indecating an incresese in Ag molf with the decreas in CuO molf in the glass networks. The increase in ion/molecule concentrations usually led to a decrease in $\hbox {R}_{i}$ and via versa^64–66^ . The results obtained in current study agrees with this behaviour (Table 4 and Fig. 7d–f). The formation of polaron resulting from the interaction between charge carriers and lattice ion vibrations greatly affect the physical properties of the hosting materials which is glass in current study^67^. By compairing the $\hbox {R}_{p}$ of the additive to the lattice constant, the polaron can either be small or large^68^ . In current study, the value of $\hbox {R}_{p}$ is inversely proportional to both CuO and Ag molfs indecating a large polaron condition and hence decreasing CuO molfs increases the stability of the formed glasee while the oposite is caused by increasing Ag molfs.

Since the value of $\hbox {n}_{b}$ depends mainly on $\hbox {C}_{no}$, $\hbox {d}_{B-B}$ and OPD^66^. The replacement of $\hbox {Cu}^{2+}$ ($\hbox {C}_{no}$ = 4) with Ag ($\hbox {C}_{no}$ = 12) may led to the increase of $\hbox {n}_{b}$ values. Generaly, The $\hbox {n}_{b}$ values increases with the decrease in $\hbox {d}_{B-B}$ and the increase in the OPD, which is not the case from CuB-1 (7.92) to CuAgB-2 (8.83). The replacement of $\hbox {Cu}^{2+}$ with Ag as stated befor may overcomed this trend. After CuAgB-2, the results of $\hbox {C}_{no}$, $\hbox {d}_{B-B}$ and OPD acted in the same direction which agreed with the found results indecating an increase from 8.83 $\times$ 10^22^ to 9.48 $\times$ 10^22^$\hbox {m}^{-3}$.

**Table 4:** Samples code and physical parameters of the glass samples.

	CuB-1	CuAgB-2	CuAgB-3	CuAgB-4
Density × 10^−1^ g $\hbox {cm}^{-3}$	23.40	23.70	23.90	24.70
$$\hbox {V}_{m}$$$$\hbox {cm}^{3}$$$$\hbox {mol}^{-1}$$	26.75	26.92	26.78	26.00
OPD g atom $\hbox {liter}^{-1}$	76.71	74.92	75.12	77.16
$$\hbox {V}_{O}$$$$\hbox {cm}^{3}$$$$\hbox {mol}^{-1}$$	13.04	13.35	13.31	12.96
$$\hbox {V}_{m}^{B}$$$$\hbox {cm}^{3}$$$$\hbox {mol}^{-1}$$	25.84	26.49	26.43	25.74
$\hbox {d}_{B-B}$ × 10^8-^ nm	3.50	3.53	3.53	3.50
$\hbox {n}_{b}$ × 10^22^$\hbox {m}^{-3}$	7.92	8.83	9.03	9.48
N × 10^20^ (Ag)	0.00	12.30	14.35	16.96
$\hbox {R}_{p}$ (Å) (Ag)	–	3.76	3.57	3.38
$\hbox {R}_{i}$ (Å) (Ag)	–	9.33	8.87	8.39
N × 10^20^ (CuO)	29.20	12.91	10.37	7.83
$\hbox {R}_{p}$ (Å) (CuO)	2.82	3.70	3.98	4.37
$\hbox {R}_{i}$ (Å) (CuO)	7.00	9.18	9.88	10.85

### Optical study of the prepared glass samples

**Figure 8:**
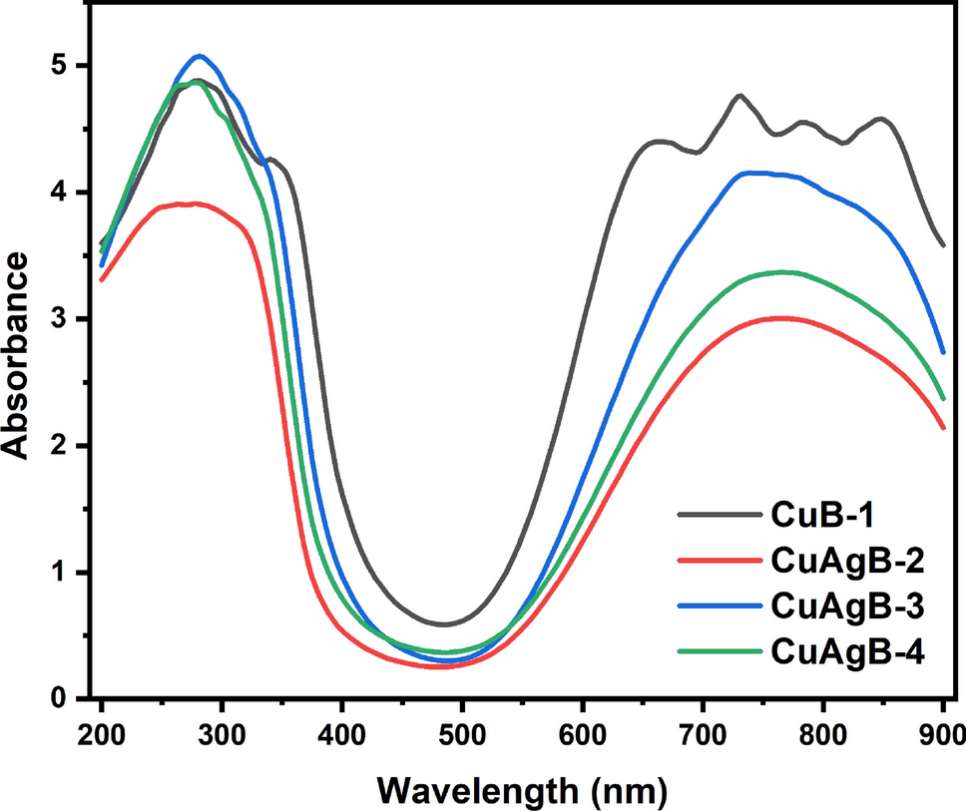
Absorbance spectra of the prepared glass samples.

Figure 8 shows the UV absorption spectra, Tauc Plot and ln($\alpha$) of all four glass samples (CuB-1, CuAgB-2, CuAgB-3 and CuAgB-4) to show how the addition of CuO/Ag nanocomposite affected the borosilicate network. Figure 8 shows that the SiEC and the Si-OHC are represented by two minor overlapping peaks at 249 and 277 nm, respectively^69^. Then, from 650 to 900 nm, there is a broad peak that is associated with copper ions. Copper oxide can be seen in absorption spectra in two different regions, each with its own unique peak. In the first region, at 240 nm, cuprous ions are visible, which overlaps with the peak at 240 nm of SiEC. In the second region, from 650 to 900 nm, cupric ions in an octahedral configuration merge into a single broad peak at 730 and 850 nm, respectively. At the highest concentration of CuO in sample CuB-1, the two peaks begin to separate. The presence of copper oxide as Cu^2+^ (d^9^) with a d-d transition is well-known. The D level splits into a (^2^E_g_) double ground state and a (^2^T_g_) triple exited state during the melting process. In addition, the levels ^2^B_1g_, ^2^A_2g_ and ^2^E_g_ are further subdivided into four levels by the Jahn-teller effect, which is described by the following wave functions: x^2^-y^2^, 3z^2^-r^2^ and XY, YZ and ZX; this effect also describes the geometrical distortion of molecules and ions caused by certain electron configurations^70^. Geometric distortions are caused by the Jahn-Teller effect, which basically applies to non-linear molecules having spatially degenerate electronic ground states. Alternatively, absorption at wavelengths below around 330 nm can be caused by ionized tiny clusters and silver ions; however, in our situation, these bands are obscured by the absorption of copper ions and silica defects, so they cannot be identified. One possible explanation for the overlapping band at 320 nm is that the produced silver nanoparticles have a surface plasmon resonance (SPR) band. The presence of an Ag band suggests that, in the case of CuAgB-4 enriched with an Ag nanoparticle ratio, the broad band’s shape extends from 200 to 335 nm, whereas in the case of CuB-1 free of Ag, the band’s shape deforms and shifts to be from 200 to 370 nm as the CuO ratio increases. The bonding formation between CuO/Ag and borate, which increases the induced defects in the borate glass network due to increasing NBO and the conversion of BO_3_ into BO_4_ groups, may be the reason for the observed increase in the intensity of the bands in the 650–900 nm range^71^, as demonstrated by the sequence CuAgB-2 $\rightarrow$ CuAgB-4 $\rightarrow$ CuAgB-3 $\rightarrow$ CuB-1. The weakening of the Si-O bond, which releases O atoms, may explain why the intensity of the bands in the 200–370 nm region changes.

### γ-ray shielding properties

**Figure 9:**
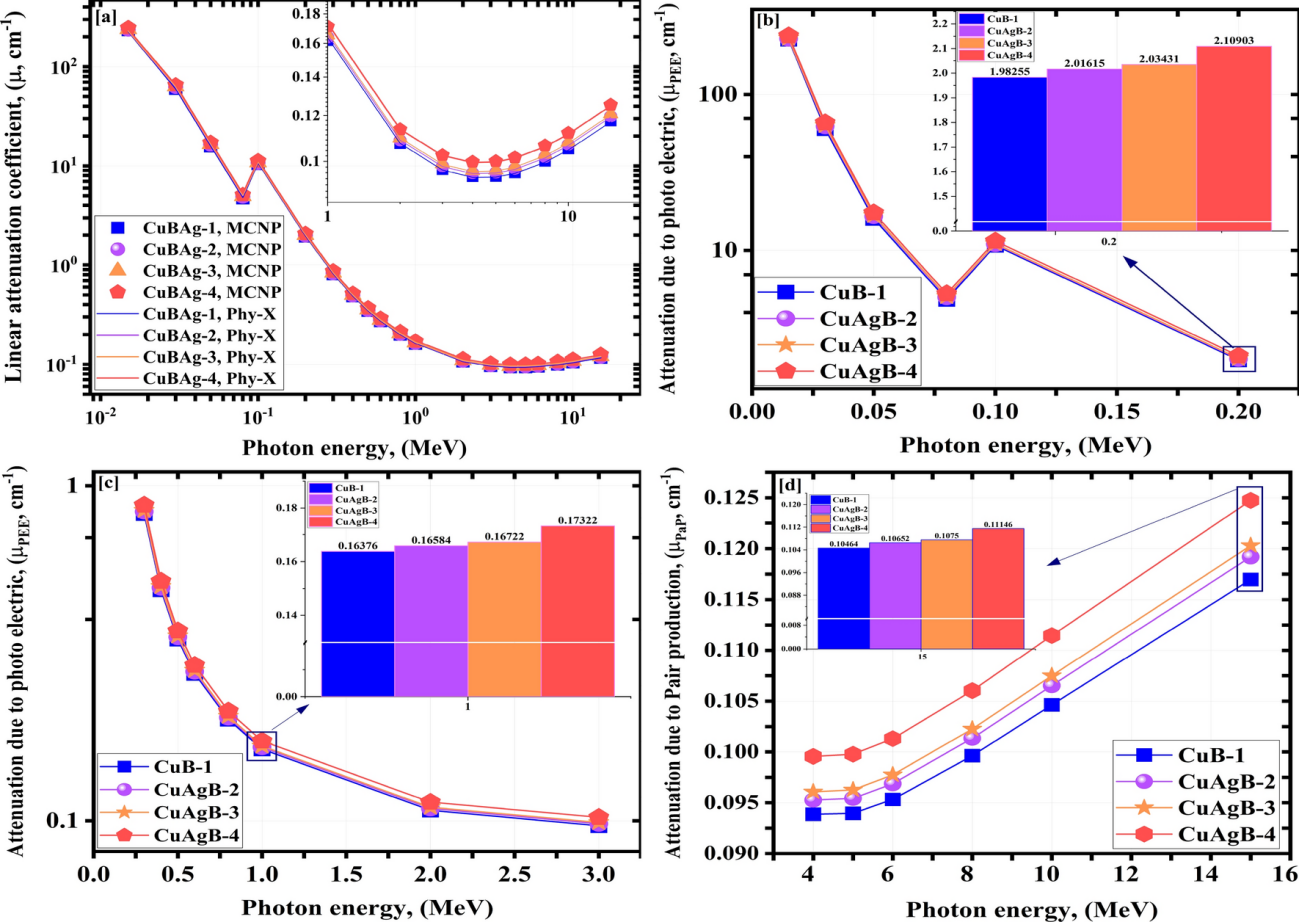
Influence of $\gamma$-ray on LAc of (**a**) obtained from Mc and PhyX, (**b**) photoelectric, (**c**) compton scattering and (**d**) pair production for the synthetic CuAgB-X glasses.

Figure 9 represents the LAc ($\mu$) of the four synthetic CuAgB-X glasses. Figure 9a represents the LAc using Mc and PhyX software in the ${\gamma }$E range of 0.15e−1: 15.00 MeV. Simulated LAc values are consistent with PhyX calculations, with a maximum $\Omega$ of 35.69e−1 $\%$, as listed in Fig. 5. The LAc of the synthetic CuAgB-X glasses decreases as the ${\gamma }$E increases. The simulated LAc values drop from 2260.23e−1 to 1.17e−1 $\hbox {cm}^{-1}$ for CuB-1, from 2278.79e−1 to 1.19e−1 $\hbox {cm}^{-1}$ for CuAgB-2, from 2296.17e−1 to 1.20e−1 $\hbox {cm}^{-1}$ for CuAgB-3 and from 2377.05e−1 to 1.25e−1 $\hbox {cm}^{-1}$ for CuAgB-4 in the ${\gamma }$E of ( 0.15e−1: 15.00 MeV).

Figure 9b showed that there is a tough decrease in the LAc for all the synthetic CuAgB-X glasses because of PEE, which has changed in the cross-section ($$\sigma$$) with $${\gamma }$$$$\hbox {E}^{-3.50:-4.50}$$. Consequently, the interaction $$\sigma$$ showed a hard decrease with the increase of $${\gamma }$$E. The increase of the applied $${\gamma }$$E of ( 0.15e−1: 2.00e−1 MeV) causes a tough exponential decreasing tendency from 2260.23e−1 to 19.83e−1 $$\hbox {cm}^{-1}$$ for the glass sample; CuB-1, from 2278.79e−1 to 20.16e−1 $$\hbox {cm}^{-1}$$ for the glass sample; CuAgB-2, from 2296.17e−1 to 20.34e−1 $$\hbox {cm}^{-1}$$ for the glass sample; CuAgB-3 and from 2377.05e−1 to 21.09e−1 $$\hbox {cm}^{-1}$$ for the CuAgB-4 glass.

Figure 9c showed that the LAc values in the $${\gamma }$$E from ( 3.00e−1: 3.00 MeV) undergo an exponential drop due to the COM interaction (changes in $$\sigma$$ caused by $${\gamma }$$$$\hbox {E}^{-1}$$). It is explained by higher-$${\gamma }$$Es’ lower propensity to interact with the material’s atoms^72^. The increase in $${\gamma }$$E was linked to a decrease in the $$\sigma$$ with drops in the quantity of photon & electron interactions, followed by a smoothy drop in the LAc. The reduction in LAc was from 8.25e−1 to 0.97e−1 $$\hbox {cm}^{-1}$$ for CuB-1, from 8.38e−1 to 0.98e−1 $$\hbox {cm}^{-1}$$ for CuAgB-2, from 8.46e−1 to 0.99e−1 $$\hbox {cm}^{-1}$$ for CuAgB-3 and from 8.77e−1 to 1.02e−1 $$\hbox {cm}^{-1}$$ for CuAgB-4 with raising the $${\gamma }$$E between (0. 300.00: 4.00 MeV).

There is a little increase due to the PaP with $\sigma$ changes with $\hbox {E}_{\gamma }^{2}$^73^. The LAc were from 0.094 to 0.117 $\hbox {cm}^{-1}$ for CuB-1, from 0.95e−1 to 1.19e−1 $\hbox {cm}^{-1}$ for CuAgB-2, from 0.96e−1 to 1.20e−1 $\hbox {cm}^{-1}$ for CuAgB-3 and from 1.00e−1 to 1.25e−1 $\hbox {cm}^{-1}$ for CuAgB-4 glass sample, raising the ${\gamma }$E between 50.00e−1: 150.00e−1 MeV as shown in Fig. 9d.

According to the previous findings, adding high amounts of silver greatly enhances the LAc of the synthetic glasses due to its high $\hbox {Z}_{ef}$ and density. The manufactured CuAgB-4 glass outperforms the other samples in terms of LAc.

**Table 5:** The LAc which was obtained via Mc and PhyX for the synthetic CuAgB-X glasses.

Photon energy (MeV)	The LAc coefficient ($\mu$, $\hbox {cm}^{-1}$)											
	CuB-1			CuAgB-2			CuAgB-3			CuAgB-4		
	Phy-X	MCNP	$\Omega$, (%)	Phy-X	MCNP	$\Omega$, (%)	Phy-X	MCNP	$\Omega$, (%)	Phy-X	MCNP	$\Omega$, (%)
0.15	2260.23	2341.97	34.90	2278.79	2359.26	34.11	2296.17	2377.75	34.31	2377.05	2468.39	37.01
0.30	601.42	599.50	− 3.20	624.32	622.37	− 3.13	631.96	629.98	− 3.15	657.31	655.09	− 3.40
0.50	159.88	157.45	− 15.43	165.92	163.45	− 15.08	167.94	165.43	− 15.17	174.67	171.86	− 16.36
0.80	48.45	47.20	− 26.46	50.16	48.90	− 25.86	50.75	49.47	− 26.02	52.77	51.33	− 28.06
1.00	107.71	105.28	− 23.11	109.68	107.26	− 22.58	110.69	108.24	− 22.71	114.79	112.04	− 24.50
2.00	19.83	19.51	− 16.04	20.16	19.85	− 15.70	20.34	20.03	− 15.79	21.09	20.74	− 17.03
3.00	8.25	8.17	− 9.69	8.38	8.30	− 9.47	8.46	8.38	− 9.52	8.77	8.68	− 10.27
4.00	4.90	4.87	− 5.68	4.97	4.94	− 5.55	5.01	4.98	− 5.58	5.19	5.16	− 6.02
5.00	3.49	3.47	− 3.86	3.54	3.52	− 3.78	3.57	3.55	− 3.80	3.70	3.68	− 4.10
6.00	2.75	2.74	− 2.84	2.79	2.78	− 2.78	2.81	2.80	− 2.79	2.91	2.90	− 3.01
8.00	2.01	2.01	− 0.84	2.04	2.03	− 0.82	2.05	2.05	− 0.83	2.13	2.13	− 0.89
10.00	1.64	1.62	− 11.52	1.66	1.64	− 11.26	1.67	1.65	− 11.33	1.73	1.71	− 12.22
20.00	1.07	1.07	− 0.96	1.09	1.09	− 0.94	1.10	1.10	− 0.94	1.14	1.14	− 1.02
30.00	0.97	0.97	1.50	0.98	0.99	1.46	0.99	0.99	1.47	1.02	1.03	1.59
40.00	0.94	0.94	2.47	0.95	0.95	2.41	0.96	0.96	2.43	1.00	1.00	2.62
50.00	0.94	0.94	2.90	0.95	0.96	2.84	0.96	0.97	2.86	1.00	1.00	3.08
60.00	0.95	0.96	2.49	0.97	0.97	2.44	0.98	0.98	2.45	1.01	1.02	2.64
80.00	1.00	1.00	4.36	1.01	1.02	4.26	1.02	1.03	4.29	1.06	1.07	4.62
100.00	1.05	1.05	4.26	1.07	1.07	4.17	1.07	1.08	4.19	1.11	1.12	4.52
150.00	1.17	1.17	4.00	1.19	1.20	3.91	1.20	1.21	3.93	1.25	1.25	4.24

All numbers are multiplied by e−1

**Figure 10:**
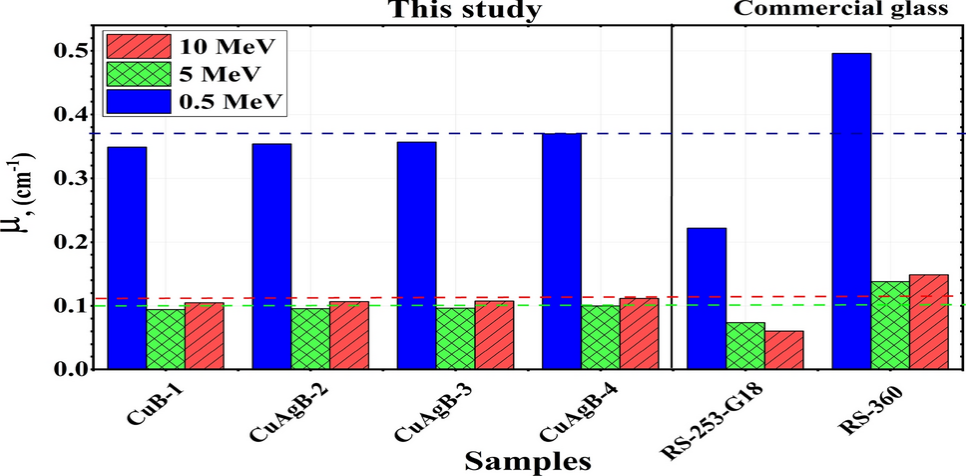
The LAc versus photon-energy for the synthetic CuAgB-X glasses compared with some reference glasses.

Figure 10 compares the LAc values of the synthetic CuAgB-X glasses and those of SCHOTT glasses (RS-253-G18 and RS-360) at chosen ${\gamma }$E of 0.50, 5.00 and 10.00 MeV^74^. At 0.50, 5.00 and 10.00 MeV, the LAc of the synthetic CuAgB-X glasses have a higher value than those of RS-253-G18 and lower than the RS-360 sample^75^.

**Figure 11:**
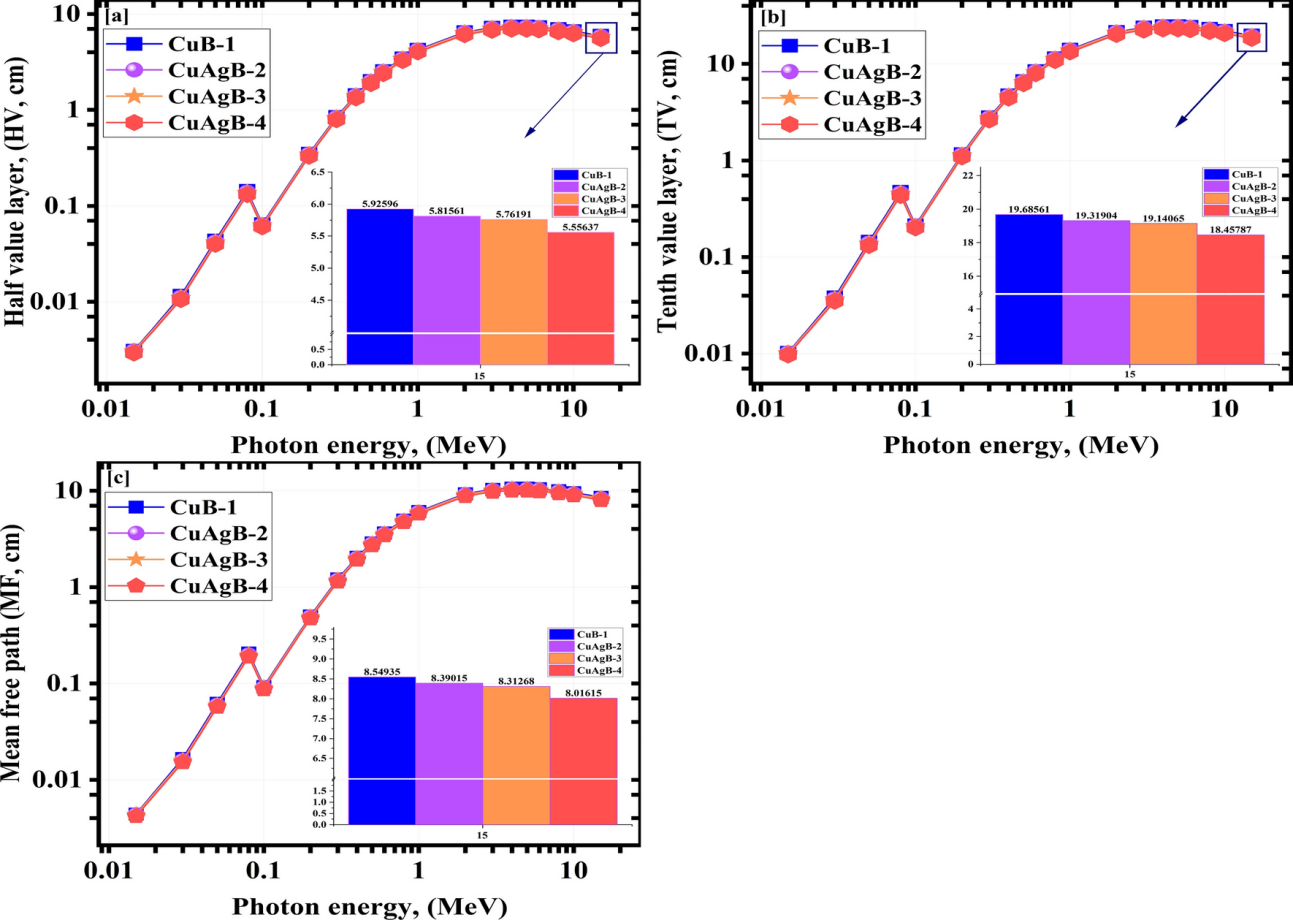
(**a**) The half value layer (HV), (**b**) The Tenth value layer (TV) and the mean free path (MF) for the synthetic CuAgB-X samples vs. the photon energy.

For a given ${\gamma }$E, high RdSg performance is often seen with a lower value for the HV, TV and MF parameters, as radiation is attenuated as it travels through a narrower zone. Typically, the HV, TV and MF all go up and down in tandem^76^. Figure 11(a,b,c) presents the values of HV, TV and MF computed by Eqs. (3), (4) and (5) . The HV of investigated CuAgB-X glasses increased as the LAc values decreased. The HV values grew from 0.03e−1 to 59.26e−1 cm for CuB-1, from 0.03e−1 to 58.16e−1 cm for CuAgB-2, from 0.03e−1 to 57.62e−1 cm for CuAgB-3 and from 0.03e−1 to 55.56e−1 cm for CuAgB-4 with raising the $\gamma$E from (0.015e−1: 15.00 MeV) as seen in Fig. 11a.

The TV values versus photon energy are illustrated in Fig. 11b.The TV’s values ranged from 0.10e−1 to 196.86e−1 cm, from 0.10e−1 to 193.19e−1 cm, from 0.10e−1 to 191.41e−1 cm and from 0.10e−1 to 184.58e−1 cm for CuB-1, CuAgB-2, CuAgB-3 and CuAgB-4, respectively.

Figure 11c represents the MF of the examined CuAgB-X glasses as it varies with ${\gamma }$E. The MF range from 184.58e−1 to 184.58e−1 cm, from 184.58e−1 to 184.58e−1 cm, from 184.58e−1 to 184.58e−1 cm and from 184.58e−1 to 184.58e−1 cm for the synthetic glasses CuB-1, CuAgB-2, CuAgB-3 and CuAgB-4, respectively.

From the obtained results, the HV, TV and MF were found to be dependent on the Nano-silver content, which indicated that the HV, TV and MF values reached the lowest values for the CuAgB-4 glass.

**Figure 12:**
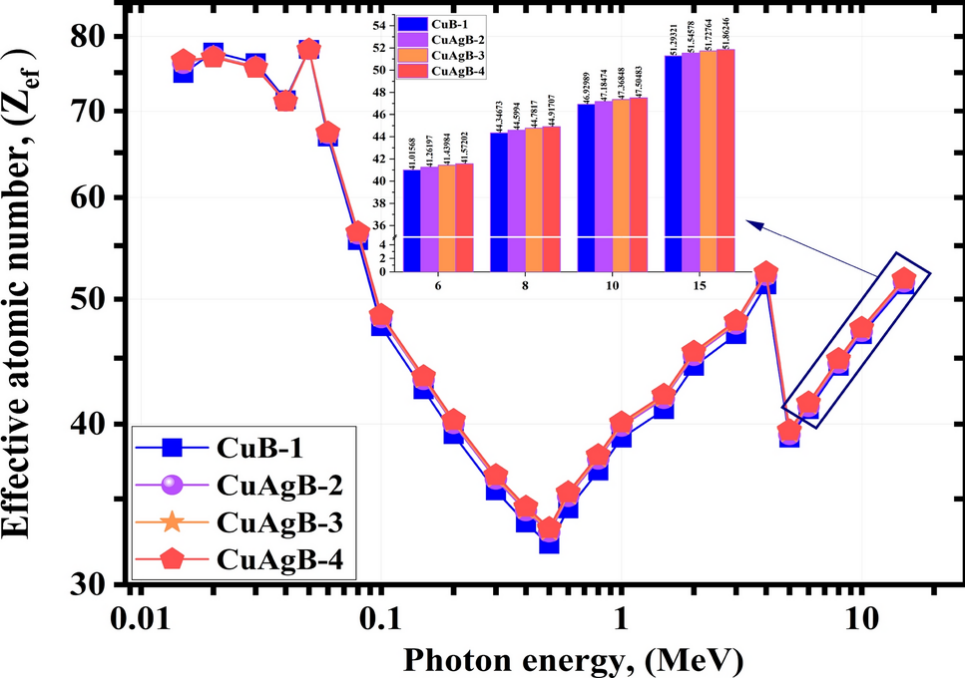
The effective atomic number (Zef) obtained from PhyX for the synthetic CuAgB-X samples vs. photon energy.

A composite’s effective atomic number, or $\hbox {Z}_{ef}$, is an important parameter with useful applications in several fields, including physics, engineering and modern technology. The increase in $\hbox {Z}_{ef}$ often indicates the enhancement in radiation interaction, especially through the COM and PEE. Consequently, high $\hbox {Z}_{ef}$-value materials may better block high-energy radiation^77^. It demonstrates that different materials may be more or less effective in attenuating radiation depending on the energy of the radiation, with higher/lower MeVs necessitating alternative materials. The range of $\hbox {Z}_{ef}$ of the synthetic CuAgB-X glasses varied from 748.92e−1–512.93e−1, 762.16e−1–521.55e−1, 764.32e−1–522.94e−1 and 766.65e−1–524.50e−1 for the synthetic glasses; CuB-1, CuAgB-2, CuAgB-3 and CuAgB-4, respectively. The $\hbox {Z}_{ef}$ explains the different characteristics of a material. The $\hbox {Z}_{ef}$ vs. the ${\gamma }$E for the investigated glasses is shown in Fig. 12.

**Figure 13:**
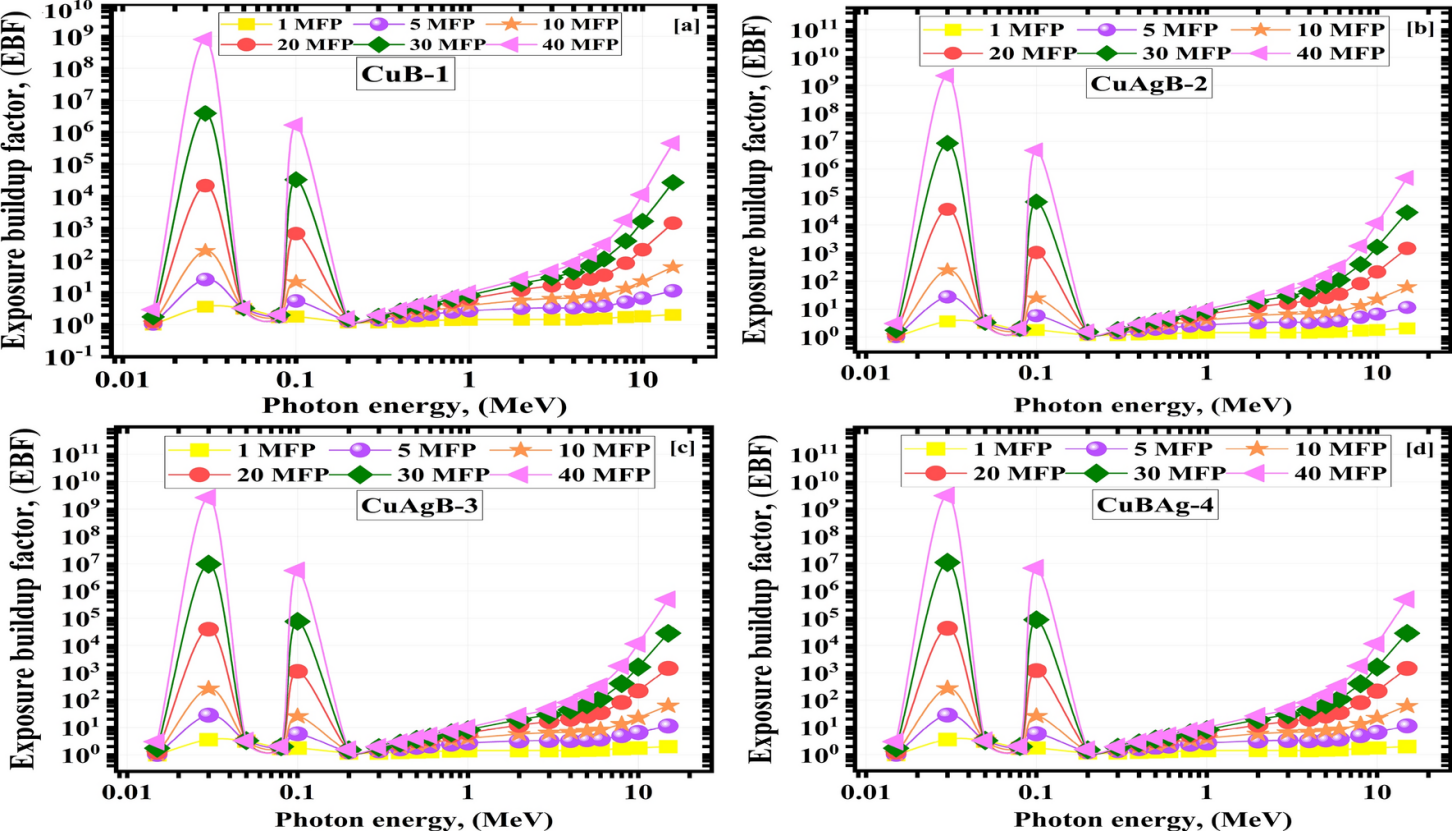
The exposure buildup factor (EBF) vs. photon energy for the synthetic CuAgB-X glass samples (**a**) CuB-1, (**b**) CuAgB-2, (**c**) CuAgB-3 and (**d**) CuAgB-4.

**Figure 14:**
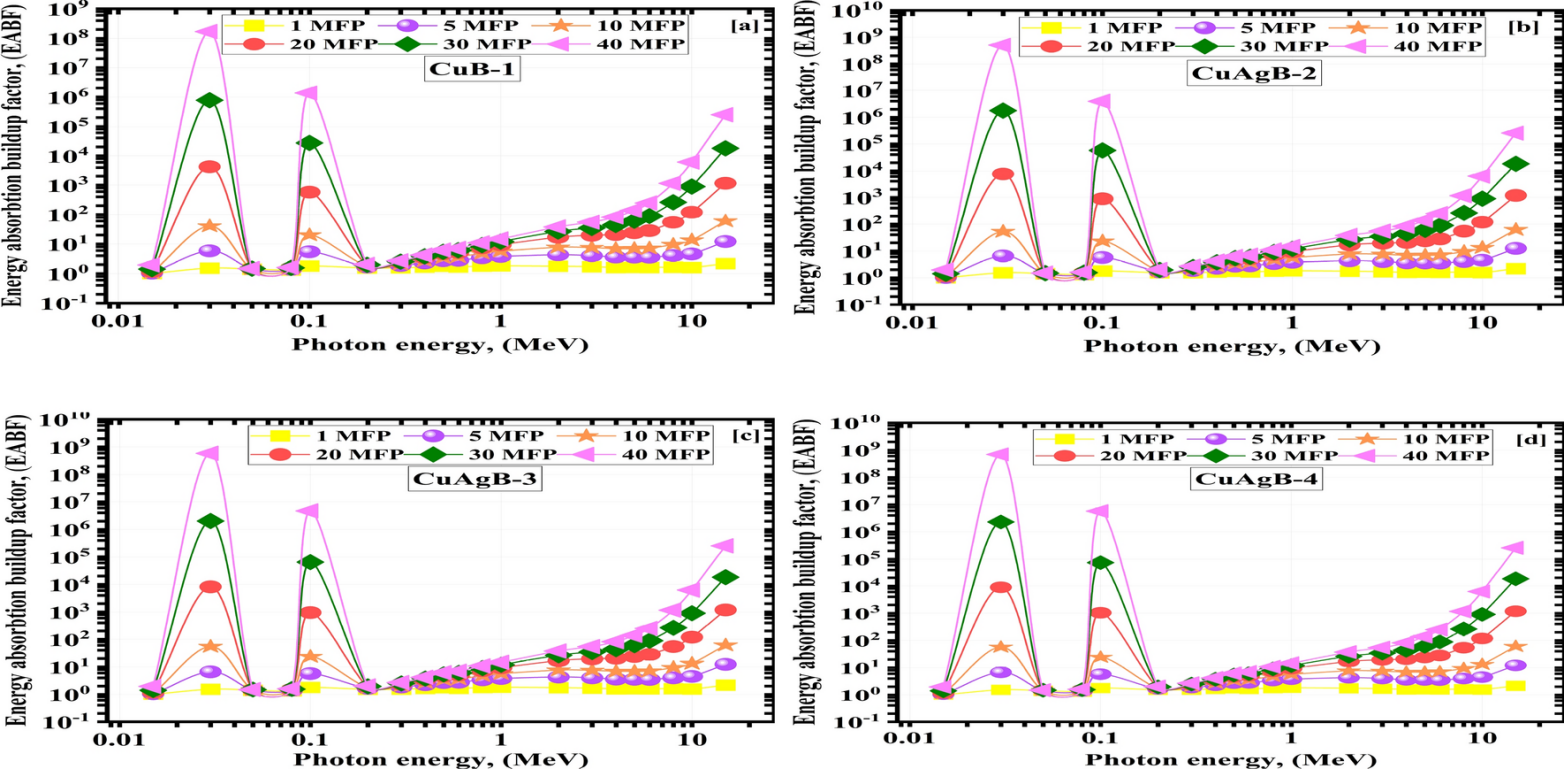
The energy absorption buildup factor (EABF) vs. photon energy for the synthetic CuAgB-X samples (**a**) CuB-1, (**b**) CuAgB-2, (**c**) CuAgB-3 and (**d**) CuAgB-4.

The energy absorption and exposure buildups (EABF and EBF) values change with photon energy for the substances being studied from 1.00–40.00 MF. They are shown in Figs. 13 and 14. Photon energy, sample composition and penetration depth all play a role in determining the upper limits of the BUFs. At larger depths, multiple scatterings occur. The 40.00 MFs penetration depth yielded the greatest BUF readings, while the 1.00 MFs depth yielded the lowest. The BUFs values increase with photon energy up to a maximum, then decrease with further increases in photon energy. Many photons have been absorbed and the BUFs are the lowest because the PEE dominates interactions at low energies^78,79^. The highest BUF values are seen in the intermediate $\gamma$E range because the prevalent COM scatters the ${\gamma }$E but cannot destroy it. The photons were again absorbed in the higher $\gamma$E area (PaP interaction).

## Conclusion

Nanocomposite samples including CuO nano-oxide and Ag/CuO nanocomposite with varying ratios were synthesized using the sonication-solgel method. The resulting samples were characterized and subsequently inserted into borosilicate glass by the melt-quenching method with the purpose of utilizing them as materials for RdSg. XPS measurements were conducted in order to investigate the properties of CuO and Ag/CuO nanocomposites. The XPS measurements provide insights into the purity of the nanocomposite samples. The analysis reveals the existence of copper in the form of Cu^2+^, as evidenced by the absence of peaks corresponding to Cu^1+^. Additionally, the XPS measurements confirm the presence of silver as $\hbox {Ag}^{0}$ inside the nanocomposite materials. X-ray diffraction (XRD) and high-resolution transmission electron microscopy (HRTEM) techniques were employed to determine the particle size of the synthesized nanocomposite. The calculation of the average particle size was performed by considering the three peaks with the highest intensity. The average particle size of CuO and Ag falls within the ranges of 29.32–35.80 nm and 35.13–45.95 nm, respectively. According to X-ray diffraction (XRD) analysis, it has been determined that copper oxide (CuO) exhibits the space group C 1 c 1 (9), whereas silver possesses the space group F m −3 m (225). X-ray diffraction (XRD) was employed to analyze the glass samples that had been doped with nanocomposite material and subjected to a melting procedure. The XRD results revealed the absence of any discernible crystalline peaks, indicating the production of an amorphous phase in the samples. Physical parameters of the glass samples were calculated including density, $\hbox {V}_{m}$, $\hbox {V}_{o}$, $\hbox {V}_{m}^{B}$, OPD, $\hbox {d}_{B-B}$, $\hbox {n}_{b}$ and N X 10^20^, $\hbox {R}_{p}$ (Å) and $\hbox {R}_{i}$(Å) for Ag and CuO. The physical properties of glass indicated an increase in density and an initial expansion in glass structural network with the addition of silver metal due to its larger size and then a compression as its molar ratio increase due to its higher $\hbox {C}_{no}$ coordination number. The electron spin resonance (ESR) technique was employed to monitor the alterations in the nanocomposites subsequent to the melting procedure within the amorphous matrix. The findings revealed the transformation of Ag^0^ into Ag^+^ and Ag^2+^ species, along with the formation of $\hbox {Ag}^{x+}_{y}$ clusters. This was evidenced by the observed g values at 2.09 and 1.980. Simultaneously, the utilization of ESR measurements allowed for the observation of CuO within the glassy matrix, manifesting as Cu^2+^ with octahedral sites. This configuration resulted in the formation of CuO^6+^ chromophores, which were interconnected through Cu–O–Cu bonds and Cu^2+^ clusters.

Also, the $\gamma$-RdSg performance of the synthetic glasses was as follows:

- Adding high concentrations of silver increases the LAc coefficient significantly. In contrast, adding it in low quantities.
- The LAc order is: CuBAg-1 < CuBAg-2 < CuBAg-3 < CuBAg-4.
- The synthesized CuBAg-4 glass sample has the lowest HV, TV and MF.
- Within the investigated energy range of $\hbox {Z}_{ef}$ within the range: from 748.92e−1–512.93e−1, 762.16e−1–521.55e−1, 764.32e−1–522.94e−1 and 766.65e−1–524.50e−1 for the synthetic samples CuBAg-1, CuBAg-2, CuBAg-3 and CuBAg-4 glasses, respectively.
- The synthesized CuBAg-4 glass sample presents the best gamma RdSg capability among the synthesized CuBAg-X glasses.

This research used lithium zinc copper borosilicate glasses as glass material and silver to make value-added products. Both materials have been synthesized and characterized to be in nanoscale to increase their gamma radiation attenuation through increasing the electron density.

## Research highlights

1. A new series of CuO/Ag nanocomposites were synthesized using sonication-solgel method (semi-green method).
2. XPS, XRD, SEM and HR-TEM were used to completely Investigate nanooxide/nanocomposites.
3. The XPS indicated high stability of silver as metal in the nano-composites; that only one sample showed some oxidation and only by 8.38%.
4. A series of borosilicate glass doped with synthesized nanooxide/nano-composites were prepared.
5. Physical, UV-Vis properties, ESR and $\gamma$-ray attenuation efficacy of the prepared glass were Investigated.
6. Physical parameters of the glass samples were calculated including density, $\hbox {V}_{m}$, $\hbox {V}_{o}$, $\hbox {V}_{m}^{B}$, OPD, $\hbox {d}_{B-B}$, $\hbox {n}_{b}$ and N, $\hbox {R}_{p}$ and $\hbox {R}_{i}$ for Ag and CuO.
7. The physical properties of glass indicated an increase in density and an initial expansion in glass structural network with the addition of silver metal due to its larger size and then a compression as its molar ratio increase due to its higher coordination number.
8. In the photon energy range of 0.15e−1 to 15.00 MeV, their gamma RdSg properties were determined theoretically using Monte Carlo simulation code and Phy-X/PSD software.
9. Finally: Nano-silver increase the gamma RdSg in the lithium zinc borosilicate glasses which could be considered a promising applicant for nuclear and medical applications.
10. Lead free glass.

## Data Availability

All data generated or analyzed during this study is included in this article.

## Abbreviations

RdSg: Radiation shielding
XPS: Chemical states analysis
ESR: Electron spin resonance
PEG: Polyethylene glycol (8000 LR)
HRs: High-resolution
PAR: Peaks area
hfs: Hyperfine structure
SiEC: Silicon electron center
Si-OHC: Silicon oxygen hole center
molf: Mole fraction
$\hbox {V}_{m}$: The molar volume
$\hbox {V}_{m}^{B}$: Boron atom molar volume
OPD: Oxygen packing density
$\hbox {d}_{B-B}$: Average boron–boron separation
$$\hbox {C}_{no}$$: Coordination number
$\hbox {x}_{B}$: Molar fraction of $B_{2}O_{3}$
Na: Avogadro’s number (602.28$\times$10^21^ g $\hbox {mol}^{-}$)
N: ionic concentration
$$\hbox {R}_{p}$$: Polaron radius
$\hbox {R}_{i}$: Inter-ionic distance
$\hbox {n}_{b}$: Bonds per unit volume
